# Coupled hydrothermal venting and hydrocarbon seepage discovered at Conical Seamount, Papua New Guinea

**DOI:** 10.1038/s41598-025-17192-x

**Published:** 2025-09-19

**Authors:** Philipp A. Brandl, Sylvia G. Sander, Christoph Beier, Mark Schmidt, Jan J. Falkenberg, Terue Kihara, Klaas Meyn, Felix Genske, Rebecca Zitoun, Brent I. A. McInnes, Mark D. Hannington, Sven Petersen, Eemu J. Ranta, Fred Jourdan, Louis-Maxime Gautreau, Thor H. Hansteen, Ingo Heyde, Stanis Konabe, Joseph O. Espi, The SO299 Shipboard Scientific Party, Octavio Acuña Avendaño, Alan T. Baxter, Christophe Y. Galerne, Max Kaufmann, Johanna Klein, Sabine Lange, Doris Maicher, Esther Panachi, Konstantin Reeck, Egor Riemer, William Ruth, Johanna Schenk, Sarima Vahrenkamp, Leon Waßmund, Julia Wenske, Hannah Zimmer

**Affiliations:** 1 GEOMAR Helmholtz Centre for Ocean Research Kiel, Kiel, Germany; 2 Faculty of Mathematics and Natural Sciences, Kiel University, Kiel, Germany; 3 Department of Geosciences and Geography, Research Programme of Geology and Geophysics (GeoHel), University of Helsinki, Helsinki, Finland; 4 GeoZentrum Nordbayern, Friedrich-Alexander University Erlangen- Nürnberg, Erlangen, Germany; 5 Department of Applied Geochemistry, Technische Universität Berlin, Berlin, Germany; 6 INES Integrated Environmental Solutions UG, Wilhelmshaven, Germany; 7 Federal Institute of Geosciences and Natural Resources (BGR), Hannover, Germany; 8 Institute of Mineralogy, University of Münster, Münster, Germany; 9 Institute for Marine and Antarctic Studies, University of Tasmania, Hobart, Australia; 10 John de Laeter Centre, Faculty of Science and Engineering, Curtin University, Perth, Australia; 11 Department of Earth and Environmental Sciences, University of Ottawa, Ottawa, Canada; 12 Western Australian Argon Isotope Facility, School of Earth and Planetary Sciences, John de Laeter Centre for Isotope Research and C-FIGS, Curtin University, Perth, Australia; 13 Earth Sciences Division, School of Natural and Physical Sciences, The University of Papua New Guinea, Port Moresby, Papua New Guinea; 14 Department of Earth Sciences, University of Toronto, Toronto, Canada; 15 Faculty of Geosciences, University of Bremen, Bremen, Germany; 16 Odyssey Marine Exploration Inc, Tampa, USA

**Keywords:** Geochemistry, Geology, Marine biology, Marine chemistry

## Abstract

During research expedition SO299 with the German RV *Sonne*, we discovered the first deep-sea hydrothermal vent system along the Tabar-Lihir-Tanga-Feni island chain in northeastern Papua New Guinea. The Karambusel vent field is hosted by a volcanic center on the western flank of Conical Seamount that formed ~ 89 ka ago. Karambusel is remarkable in that it hosts both a fossil high-temperature, gold-rich mineralization and an active low-temperature (< 51 °C) vent system precipitating arsenic-, antimony-, thallium-, and mercury-rich sulfide minerals. Chemosymbiotic fauna is associated with the vent system and we identified more endemic species than in previous studies on nearby seeps. Our study shows that the magmatic event at Karambusel likely triggered the epithermal mineralization at Karambusel and at the central summit of Conical Seamount. The current hydrothermal fluids originate from condensed magmatic vapor or connate fluids. Gas bubbles were observed at some vent sites and the proportion of methane in the gas phase exceeds that of any other hydrothermal vent system. The composition of the light hydrocarbons points towards a thermogenic origin. Karambusel is thus the first hybrid magmatic-hydrothermal vent and hydrocarbon seep system discovered globally which explains the highly endemic vent fauna as a consequence of the unique ecological niche.

**Supplementary Information:**

The online version contains supplementary material available at 10.1038/s41598-025-17192-x.

## Introduction

**Fig. 1:**
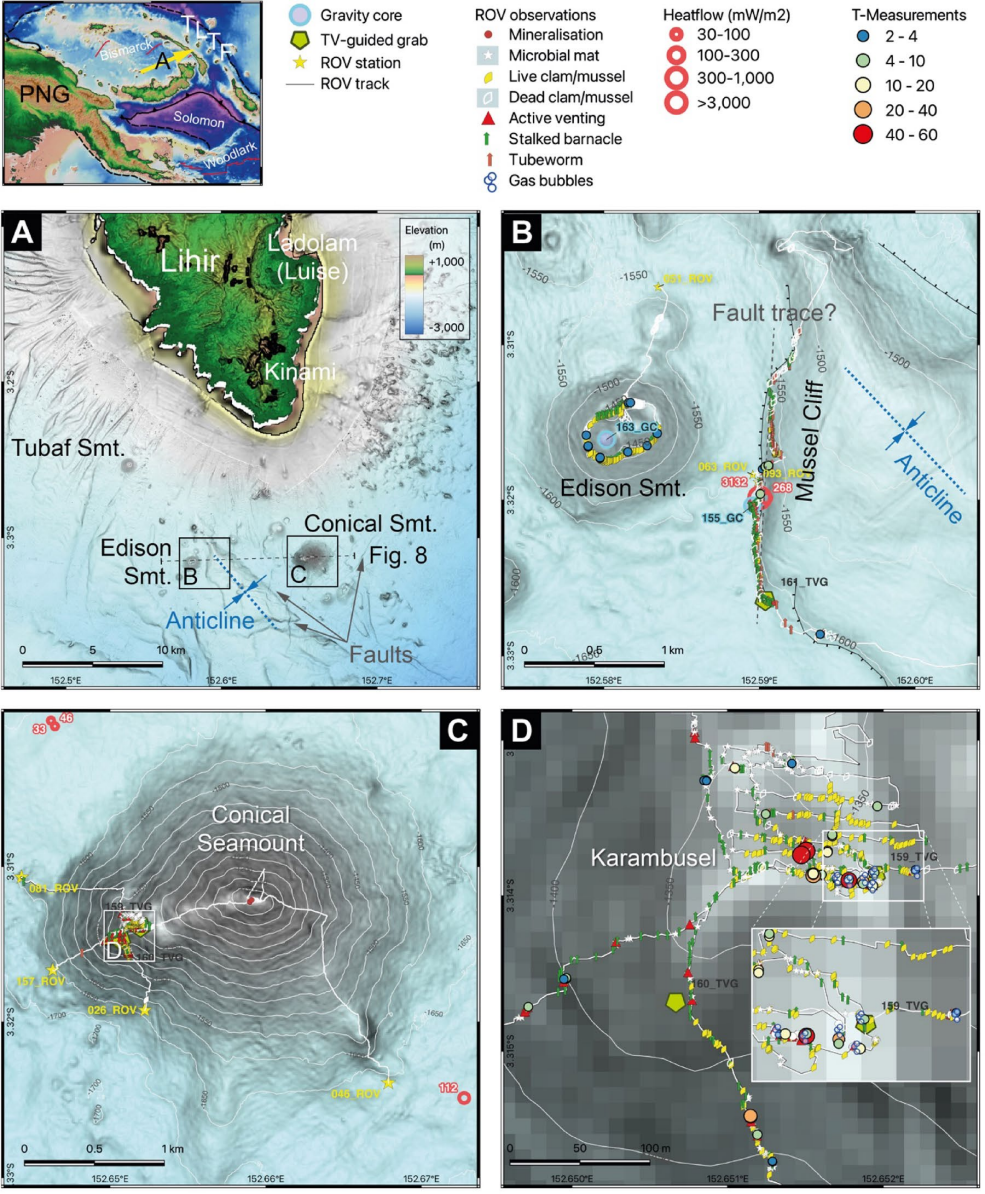
Bathymetric maps of the sites investigated. The small map at the top illustrates the locality of Lihir island (arrow) within the TLTF island chain in Papua New Guinea (PNG). (**A**) Overview map of the study area with the southern part of Lihir Island including the Luise (hosting the Ladolam Au mine) and Kinami volcanic complexes and the offshore South Lihir Volcanic Field; Note the faults around Conical Seamount and the anticline SE of Edison Seamount. The approximate position of profile shown in Fig. 8 is illustrated by the black dashed line. The blue dashed line depicts the axis of the seismically imaged anticline. (**B**) Map of Edison Seamount and Mussel Cliff and (**C**) Conical Seamount along with SO299 stations, ROV tracks, observations and measurements. (**D**) Close-up of the Karambusel vent field. Maps created with QGIS 3.34 LTR using bathymetric data of RV Sonne expeditions SO94, SO133, SO166 and SO299, and ASTER Global Digital Elevation Model V003 topographic data (NASA/METI/AIST/Japan Spacesystems and U.S./Japan ASTER Science Team).

The ~ 250 km-long Tabar-Lihir-Tanga-Feni (TLTF) island chain in northeastern Papua New Guinea (Fig. 1) is a hot spot for oceanographers, biologists, geologists, and the mining industry. Its individual islands represent the emerged portions of volcanic complexes that formed on structural highs within the New Ireland Basin^1^. The basin started to form in a forearc setting in the late Eocene some 40 Ma ago and since then accumulated up to 6 km of volcaniclastic and carbonate sediments e.g.,^1,2^. Today, the basin is undergoing extension in NW-SE direction and compression in NE-SW direction. Crustal thinning triggered alkaline magmatism 3.6 Ma ago and volcanism is decreasing in age towards New Ireland^1^.

Some volcanic systems in the TLTF island chain are host to porphyry and epithermal gold mineralization including the active mines at Simberi in the Tabar island group and Ladolam (Luise volcano) on the island of Lihir (Niolam), and the Conical Seamount, ~ 20 km to the south e.g.,^1,3^ (Fig. 1). Ladolam is exceptional in being the world’s largest alkalic low-sulfidation epithermal Au deposit in terms of total contained Au (50 Moz or > 1,400 t)^4^. No signs of recent hydrothermal activity were observed at Conical Seamount during research expeditions SO94 (1994), SO133 (1998) and SO166 (2002) with the German RV *Sonne*, and FR04/00 (2000) with the Australian RV *Franklin*. Chemosymbiotic fauna was observed, collected, and described at two other localities in the TLTF chain that are < 10 km east of Conical Seamount and are less than one kilometer apart from each other: Edison Seamount and the so-called Mussel Cliff (Fig. 1A). The fauna collected from these two localities e.g.,^5–7^ shows little taxonomic connectivity to other vent systems in the western Pacific^8^. This contrasts with the hydrothermal vent fauna of the nearby Manus backarc basin (part of the ‘Bismarck Sea’; Fig. 1) that acts as a network hub for the western Pacific vent fauna^8^.

In addition, no active deep-sea hydrothermal vent sites were previously known from the TLTF island chain. The presence of chemosymbiotic fauna was interpreted to result from widespread diffuse hydrothermal flow at the volcanic Edison Seamount^9^. Strong methane (CH_4_) water column anomalies (0.5–10 µL L^− 1^), with highly negative carbon isotopic compositions (δ^13^C < -50‰) of hydrocarbons and the presence of authigenic carbonate minerals led to the conclusion that Mussel Cliff is an active cold seep^10^.

In this study, we report the discovery of the first active deep-sea hydrothermal vent field in the TLTF island chain. Seafloor footage, samples, and data were collected during SO299 DYNAMET (geoDYNAmics & METallogeny) with the German RV *Sonne* in June-July 2023 using the Remotely Operated Vehicle (ROV) *Kiel 6000*. The newly discovered Karambusel vent field represents a unique site combining features of hydrothermal venting and hydrocarbon seepage at a single locality. The site records a multi-stage mineralization history and is characterized by the simultaneous expulsion of warm hydrothermal fluids (max. 51 °C) and cooler (< 20 °C) hydrocarbon-rich fluids and gases of thermogenic origin. The vent field is situated at the top of a younger volcanic edifice that erupted at the western flank of the 287 ka-old^1^ Conical Seamount. The seamount formed on top of a 3–4 km (locally exceeding 6 km) thick sequence of volcaniclastic and carbonaceous sediments that accumulated in the basin since the Eocene^1,2^. Thus, multiple host rock lithologies may influence the fluid and gas compositions and as such, the vent field and associated mineralization combine features of different types of ore deposits (epithermal, hydrothermal, and sediment-hosted). Our new findings of vent specific fauna creates additional links to other western Pacific vent systems and therefore may limit the high level of system endemism recently suggested for this area^8^.

## Results and discussion

### Discovery of the Karambusel vent field

Repeated marine scientific surveys did not find conclusive evidence for active hydrothermal venting at Conical Seamount. However, commercial exploration activity by the Bismarck Mining Corporation (PNG) Ltd., and after the acquisition of the exploration license (EL1877), by Odyssey Marine Exploration Inc. (OMEX), Tampa, USA, revealed the presence of two potential hydrothermal plumes, one above (960 m below sea level (mbsl)) and one slightly below (1,070 mbsl) the top of Conical Seamount at 1,055 mbsl. Our new high-resolution ship-based bathymetry (10 m grid) revealed structures at the western flank of Conical Seamount that we interpret to represent flank eruptions (Fig. 1C).

Here, GEOMAR’s ROV *Kiel 6000* was deployed for its first dive during this expedition (station 026) on 15th June 2023 (local time). While diving up the flank towards the summit plateau, we discovered a new vent field with extensive bacterial mats and mussel beds, and multiple vents expelling shimmering fluids and free gas (Fig. 1C, D). The vent field was visually mapped and sampled for rocks, fluids and gases (stations 026, 081 and 157). Additional rock sample material was recovered with two video-guided grabs (stations 159 and 160) (‘TVG’; Fig. 1D). The vent field was named ‘Karambusel’ (‘mussel’ in the local Tok Pisin language) after the extensive mussel beds present and in accordance with the local observer Stanis Konabe. The respective volcanic edifice on the western flank of Conical Seamount was named the Karambusel eruptive center.

**Fig. 2:**
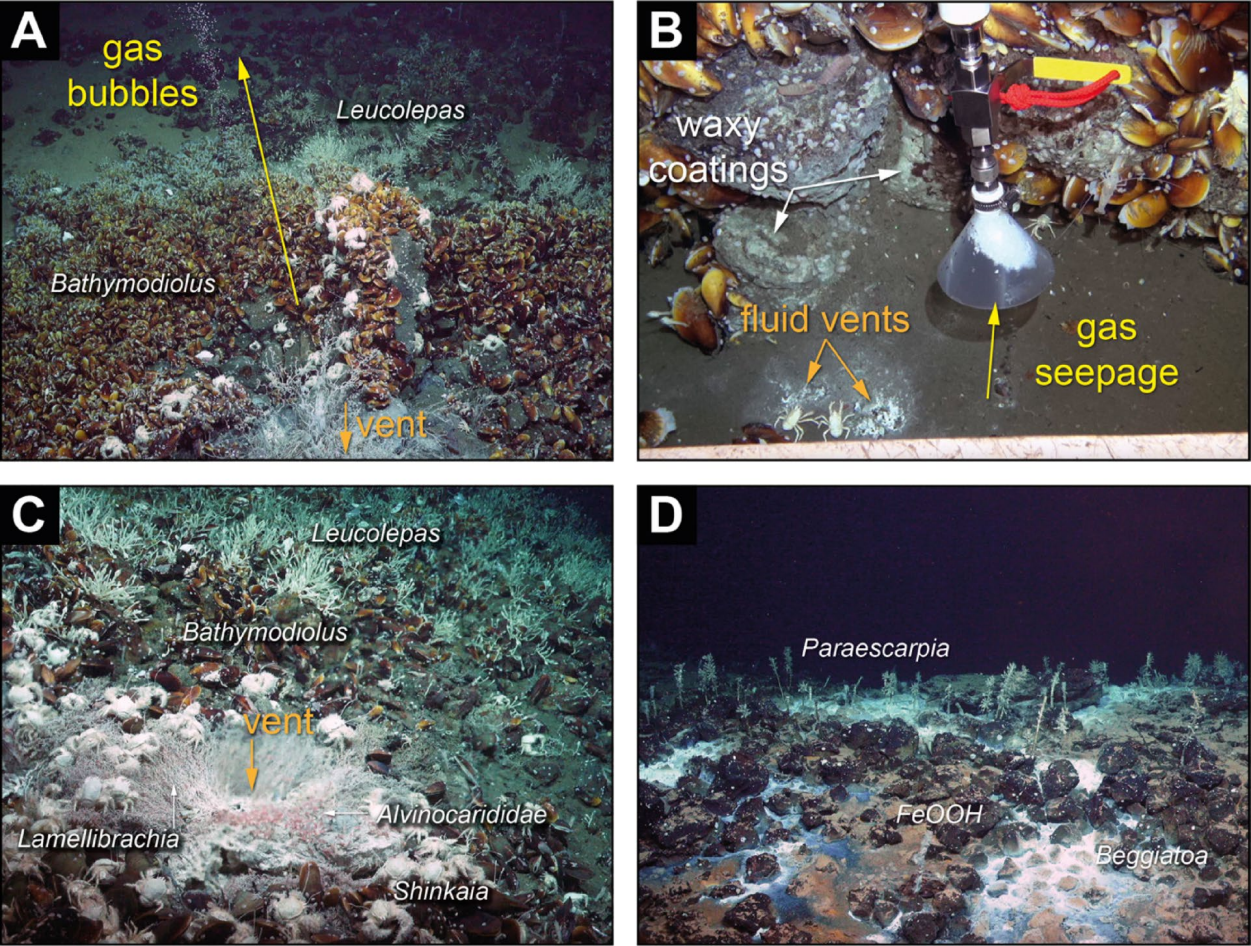
Representative ROV images of the Karambusel vent field. (**A**) Focused fluid and gas discharge (between samples 026_ROV-07 and -08). (**B**) Juxtaposition of a 41.9 °C warm fluid vent (no gas) and a cooler fluid vent with gas bubbles (10.2 °C) (081_ROV-13); Note the absence of fauna in direct vicinity of the vents. (**C**) Shimmering warm fluids (51 °C) discharging from a focused vent site. Note the abundant tube worms, shrimps and crabs close to the vent, *Bathymodiolus* mussels in proximal and stalked barnacles in distal position to the site (157_ROV-10). (**D**) Patches of white microbial (*Beggiatoa* gen. inc.) mats and dispersed tube worms *Paraescarpia echinospica* sp. inc. within volcanic rocks stained with iron oxyhydroxide minerals (081_ROV-19).

Chemosymbiotic faunal communities and discharge sites are distributed over the upper southern and western flanks of the Karambusel eruptive center, its summit area and the saddle between Karambusel and the Conical main edifice. The vent field extends approximately 300 by 300 m at a water depth between 1,315 and 1,460 mbsl (Fig. 1D). Fluid-rich vents, commonly associated with rubble piles and shimmering water, record temperatures up to 51 °C relative to ambient seawater (2.6 °C) (Fig. 2C). The highest temperatures and most focused fluid discharge sites are concentrated at the southern rim of the Karambusel summit plateau (Figs. 1D and 2C). Multiple streams of gas bubble discharge were observed at this part of the vent field (Fig. 2A, B) and water column acoustic anomalies (‘flares’) were imaged for up to 400 m above the seafloor. In one exceptional case, a gas bubbling site (~ 10 °C) was found at a short distance of only 0.3–0.4 m to a vent discharging 42 °C warm fluids (Fig. 2B). However, no fluids containing sulfide colloids, and no chimneys or mounds of hydrothermal precipitates were observed.

### Young volcanic rocks host Karambusel

**Fig. 3:**
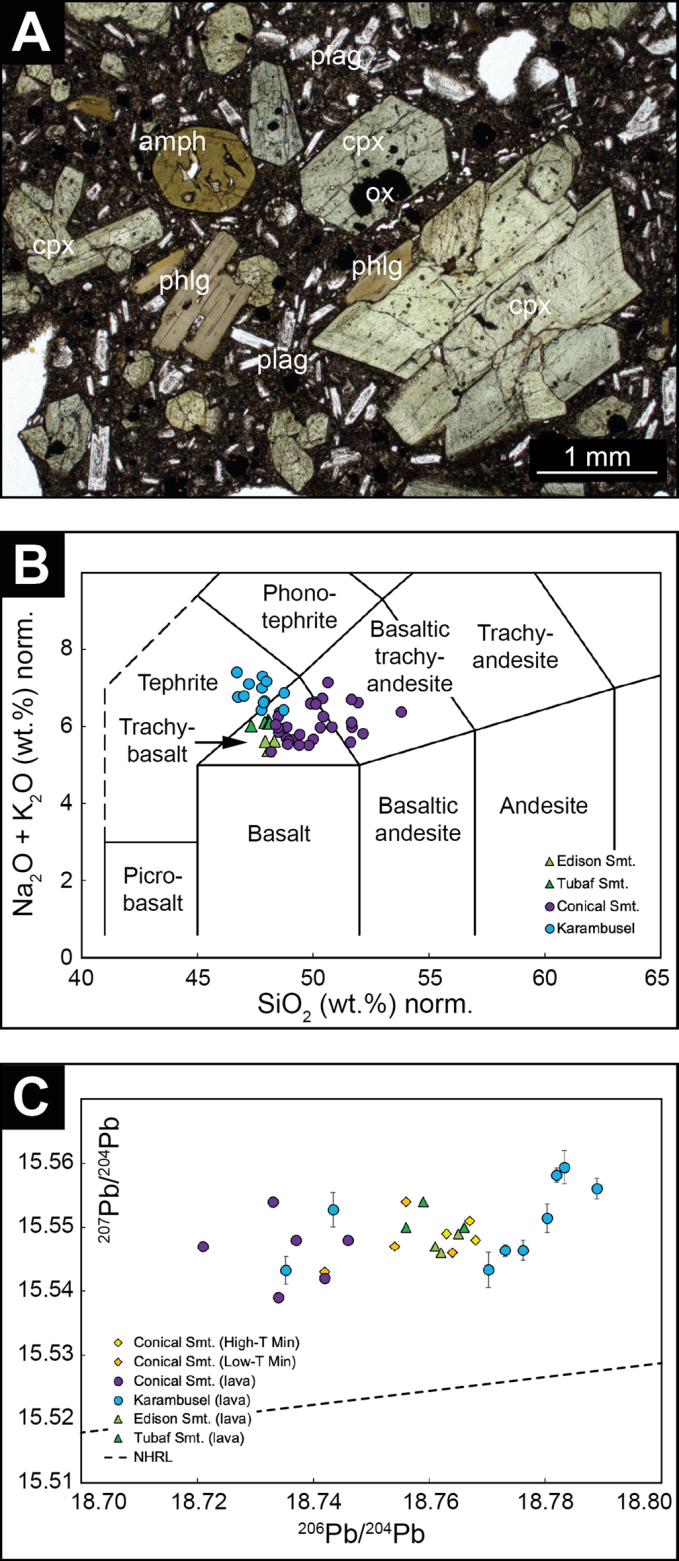
Petrology and geochemistry of unaltered volcanic whole rock samples (loss on ignition < 3 wt%). (**A**) Polarized plain light microphotograph of sample 157_ROV-17 from Karambusel: amph – amphibole, cpx – clinopyroxene, ox – oxide, phlg – phlogopite, plag – plagioclase. (**B**) Geochemical data of unaltered lavas from Conical, the nearby Edison and Tubaf seamounts^11–14^ (Fig. 1) and the Karambusel eruptive center in the total alkali versus silica diagram^15^ and in (**C**) ^206^Pb/^204^Pb versus ^207^Pb/^204^Pb. Lead isotope data of Karambusel (lava) from this study and all other data from Kamenov et al.^12^. NHRL – Northern Hemisphere Reference Line after Hart^16^.

The morphology of the Karambusel eruptive center and the visual observations indicate that this edifice represents the product of a younger volcanic event than that forming the Conical main cone. Unaltered volcanic rock samples collected from and in the vicinity of the Karambusel vent field are clinopyroxene–amphibole–phlogopite phyric mafic volcanic rocks (Fig. 3A) that differ from those at the Conical main cone, which are clinopyroxene phyric only^11^. A detailed petrographic and geochemical study of the Karambusel lavas is currently in progress and beyond the scope of this contribution. First results from geochemical analyses show that Karambusel lavas are mainly tephrites and as such are more silica undersaturated (normalized SiO_2_ < 49 wt%) but more alkaline (up to 7.4 wt% K_2_O + Na_2_O) than the lavas from the Conical main edifice^11–14^ (Fig. 3B; Tab. S1). Lead isotope analyses yield more radiogenic ^206^Pb/^204^Pb (> 18.77) in Karambusel (Tab. S2) than in Conical Seamount lavas (< 18.75) except for two samples recovered from the lower slopes of the Karambusel eruptive center (Fig. 3C). We interpret the small but significant Pb isotopic difference to reflect different magma batches. The presence of hydrous (i.e. hydroxyl group) phenocryst phases such as amphibole and phlogopite in the Karambusel lavas further indicate higher magmatic water activities. Argon (^40^Ar/^39^Ar) age dating of amphibole extracted from two mafic volcanic rock samples at the foot of the Karambusel eruptive center return two statistically indistinguishable plateau ages of 90 ± 4 ka (026_ROV-01) and 91 ± 9 ka (26_ROV-03). The age obtained from phlogopite extracted from sample 026_ROV-03 provides a higher precision and is considered to represent the best estimate of the age of eruption at 88.5 ± 0.9 ka. Please see the methods section and Fig. S3 for details.

### Fossil Au-rich mineralization at Karambusel

No seafloor massive sulfides such as chimneys or mounds, commonly found at high-temperature hydrothermal sites, are evident at the Karambusel vent field^17^. However, the volcanic rocks at Karambusel show variable hydrothermal alteration including propylitic-like (quartz + albite + illite ± adularia) and advanced argillic (quartz + pyrophyllite ± kaolinite ± dickite) alteration assemblages cf.^18^ associated with sulfide mineralization. Gold and Ag contents of up to 28 and 2,400 µg g^− 1^, and Ag to Au ratios > 6 were measured in bulk rock samples. The Au grades at Karambusel are lower than at Conical Seamount (26 µg g^− 1^ as reported by Petersen et al.^3^) even though this observation may be biased by fewer samples available from Karambusel. Arsenic contents at Karambusel (> 1 wt%) are comparable to Conical Seamount, whereas Ag and Sb (> 5,800 µg g^− 1^) contents are almost doubled, and Hg (< 500 µg g^− 1^) contents almost tripled (table S4).

**Fig. 4:**
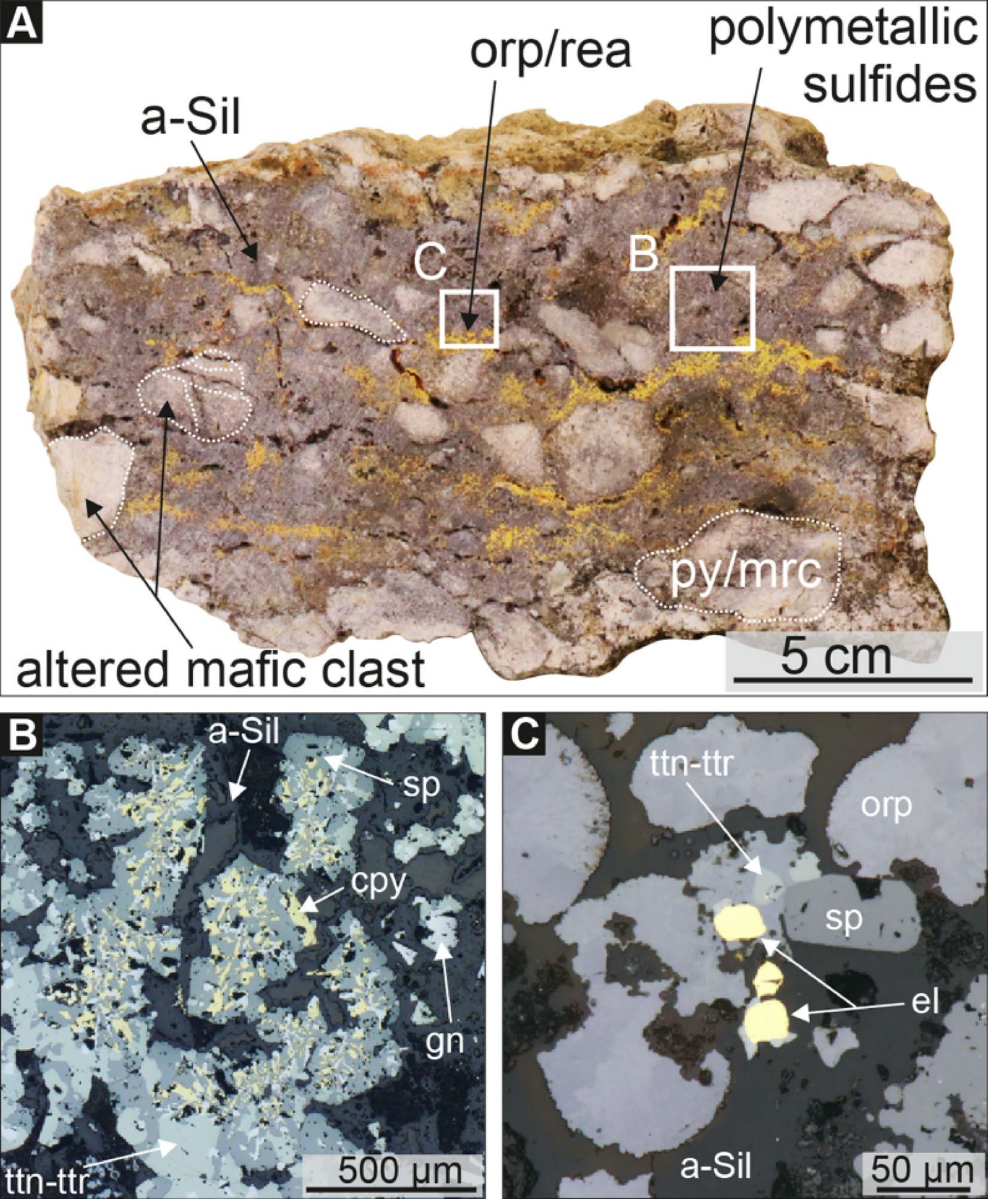
Specimen and reflected light microscopic images of the Au-rich polymetallic breccia. (**A**) Polymetallic breccia with pyrite/marcasite (py/mrc) bearing mafic clast embedded in a sulfide-rich amorphous silica (a-Sil) matrix overprinted by orpiment (orp). (**B**) Dendritic intergrowth of sphalerite (sp), chalcopyrite (cpy), and galena (gn). (**C**) 20 μm sized electrum associated with tennantite-tetrahedrite (ttn-ttr), sphalerite, orpiment, and amorphous silica.

Our petrographic examination reveals high temperature mineralization preceding the mineralization associated with the recent low temperature venting. The first mineralization stage initiated with a brecciation event and is characterized by polymetallic and Au-rich vein-style mineralization (Fig. 4A) similar to the epithermal mineralization known from the top of Conical Seamount^3^. In these breccias, dendritic intergrowth of chalcopyrite, sphalerite, and galena (Fig. 4B) indicates co-precipitation of Cu, Zn, and Pb. We interpret these sulfide mineral textures to reflect abruptly changing fluid conditions typical for fluid boiling similar to observations from the Nifonea hydrothermal field^19^ and the artificial vents in the Iheya-North vent field^20^.

At 1,350 mbsl, the boiling curve of seawater is intersected at ~ 340°C^21^ providing a minimum fossil fluid temperature consistent with the precipitation of high-temperature sulfide minerals such as chalcopyrite. Other key phases are electrum (Au-Ag alloy) with up to 20 μm grains (Fig. 4C) and As-, Sb-, Ag- and Pb-rich sulfosalts such as tennantite-tetrahedrite (Cu_12_[As, Sb]_4_S_13_), zinkenite (Pb_9_Sb_22_S_42_), famatinite (Cu_3_SbS_4_), seligmanite (PbCuAsS_3_) and stephanite (Ag_5_SbS_4_). Based on mineral assemblages of different sulfosalt and base-metal sulfide minerals, the epithermal-style Au-rich polymetallic breccia likely formed at an intermediate sulfidation state condition^22^ which contrasts the low sulfidation state of the nearby Ladolam Au deposit^4^.

Textural evidence suggests a subsequent overprint by a lower temperature stage (Fig. 4A) consisting of native sulfur as crusts, pore fillings and waxy coatings (associated with active venting; Fig. 2), minor framboidal pyrite, As- and Sb-rich sulfide minerals such as orpiment (As_2_S_3_), realgar (As_4_S_4_), stibnite (Sb_2_S_3_), and Hg- and Tl-rich sulfide minerals (e.g., cinnabar) precipitating in an amorphous silica matrix. This mineral assemblage indicates temperatures of 25 to < 100°C^23^ – a range consistent with in situ fluid temperatures.

### Low temperature active venting at Karambusel: discharge of fluids and gases

**Fig. 5:**
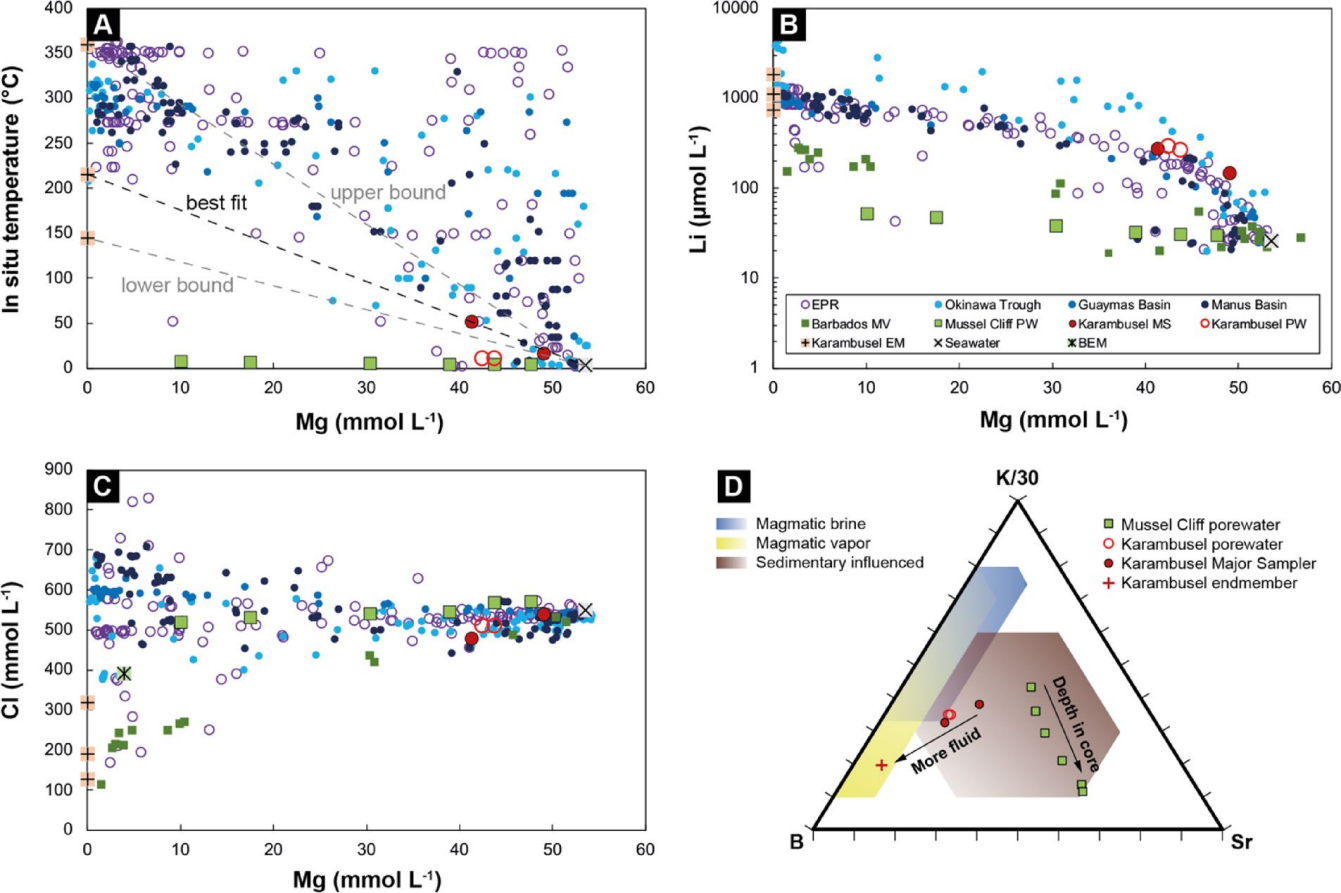
Composition of fluids (*Major Sampler* – MS) and porewaters (PW) collected at the Karambusel vent field and Mussel Cliff compared with literature data of the East Pacific Rise (sediment-free mid-ocean ridge), the Okinawa Trough (sediment-rich continental backarc basin), the Guaymas Basin (sediment covered mid-ocean ridge), the Manus Basin (backarc basin) to the opposite site of New Ireland (MarHys database of Diehl & Bach^27^ in version 4.0), and mud volcanoes (MV) of the Barbados accretionary complex^28,29^. Karambusel EM – Fluid endmember interpolated from fluids collected by *Major Samplers*, BEM – Barbados endmember of Dia et al.^29^. All elements are plotted versus Mg (mmol L^− 1^). (**A**) Measured in situ fluid temperature (in °C) versus Mg (mmol L^− 1^); Note that the temperature of the Mussel Cliff samples (155_GC) was reconstructed from in situ measurements by the heatflow probe at the same locality as the gravity corer, (**B**) Li (µmol L^− 1^) versus Mg (mmol L^− 1^), note the logarithmic scale of Li, (**C**) Cl versus Mg (both in mmol L^− 1^), and (**D**) Ternary plot of K (divided by a factor of 30), B and Sr illustrating the different fields for a magmatic brine (blue), magmatic vapor after phase separation and contraction (yellow) and for fluids that interacted with sediments (brown) after Large et al.^30^; Note the different trends in porewater from Mussel Cliff and the fluids from the Karambusel vent field.

Fluids were collected at the Karambusel vent field from 10 different sites using *Major Samplers* and *Niskin* bottles. Two porewater samples extracted from a push core are similar in composition to the fluids collected directly from vents (Tab. S5). The Karambusel fluids are mildly acidic (pH ~ 5.9), have increased total alkalinity (≤ 14.8 meq L^− 1^), Si (≤ 1.00 mmol L^− 1^) and Li (≤ 288 µmol L^− 1^) concentrations, relative to seawater (pH 7.8; 2.3 meq L^− 1^, 0.01 mmol L^− 1^ and 26 µmol L^− 1^, respectively)^24^. Linear trends in plots of Mg versus other elements indicate mixing of seawater with a hydrothermal fluid and can be used to infer the fluid endmember composition^24^. Despite our efforts of sampling the fluid endmember directly in the vents, high Mg contents of ≥ 41.4 mmol L^− 1^ indicate that no more than 23% of this fluid could be sampled if we assume an endmember Mg concentration of 0. The chemical trends for fluids at Karambusel are different from Mussel Cliff and Edison Seamount, where chemosymbiotic fauna were described previously^5–7,25,26^ (Fig. 5). The low proportion of hydrothermal fluid relative to seawater induces a relatively large uncertainty on the linear interpolation to the potential endmember composition. Endmember fluid temperatures, for example, may range from 145 to 360 °C when accepting an uncertainty of ± 10% in the elemental concentration of the purest fluid sample, with a best fit at ~ 215 °C (Fig. 5A). This interpolated fluid endmember has very high Li (736–1834 µmol L^− 1^; Fig. 5B) and B (5.89–14.7 mmol L^− 1^) at intermediate B/Li, lower Cl (128–319 mmol L^− 1^; Fig. 5C) at an elevated Na to Cl ratio of 1.21, high Si (1.78–4.45 mmol L^− 1^), but low Ca (3.80–9.47 mmol L^− 1^) and Sr concentrations (67.3–168 µmol L^− 1^; Tab. S5).

High Li contents of > 350 µmol L^− 1^ and intermediate B to Li ratios of 1–30 at Karambusel are common characteristics of sediment covered hydrothermal systems and cold vents with a high temperature (> 150 °C) imprint^31^. Endmember-corrected Cl contents are significantly lower than ambient seawater (559 mmol L^− 1^), which may indicate phase separation and subsequent condensation of the vapor phase^32^. However, in situ vent temperatures are ~ 290 °C lower than the seawater boiling temperature at the depth of the vent field. In the modern Karambusel vent system, boiling is thus restricted to the subseafloor as no vigorous “flame-like” jets of water vapor indicative for fluid boiling e.g.,^33^ were observed at the seafloor. Such ‘vapor phase’ low chlorinity fluids are commonly enriched in volatile elements such as As, Sb, Tl and Hg e.g.,^34^. Therefore, we conclude that subseafloor boiling could contribute to the volatile element-rich mineralization associated with the active venting.

Some fluid vents are also emitting gas bubbles and exclusively occur along a ~ 60 m-long section with an E-W orientation at the southeastern summit plateau of the Karambusel edifice (Fig. 1D). These vents are characterized by lower fluid temperatures of 3–16 °C but two sites show higher temperatures of 23.6 and 41.9 °C. However, the site with the 41.9 °C fluid temperature shows a clear physical separation of the warm fluid and gas-rich venting (10.2 °C), both of which are only ~ 0.3 m apart (Fig. 2B). We successfully sampled the gas streams at three different sites using gas-tight funnel samplers even though the funnel rapidly clogged up by methane clathrates (Fig. 2B). The gas samples were analyzed by gas chromatography and mass spectrometry (GC-MS) at GEOMAR. Results of gas composition analyses including their short-chain alkane and carbon isotopic composition are reported in Table 1 (dry gas samples) and S6 (headspace samples). In this contribution, we focus on the low molecular weight hydrocarbons (C_1_-C_5_) but it is important to report that longer chain hydrocarbons (C_5+_) including unsaturated hydrocarbons and polycyclic aromatic and phenolic compounds are also present in these samples.

**Table 1:** Karambusel vent field dry gas compositional data (in vol% at atmospheric pressure and room temperature). Stable carbon isotope composition δ^13^C = (((^13^C/^12^C)_sample_/(^13^C/^12^C)_standard_-1)*1000) is given in ‰ relative to the Vienna-Pee Dee belemnite (V-PDB) reference material. Reference values provide the general composition of wet natural gas after Speight^35^ the gas proportion of the Ladolam geothermal fluid sampled at the GW02 well after Simmons & Brown^36^ and vent gas sampled at the Calypso South vents offshore new Zealand (SO135, station 108) after Botz et al.^37^.

Component	Karambusel vents			Reference values		
	081_ROV-22_w	81_ROV-13_g	157_ROV-13_b	Natural gas (wet)	Ladolam geothermal fluid (GW02)^#^	Calypso South, vent gas (SO135)
Methane (C_1_)	81.16	80.74	82.75	92.46	0.71	9.898
Ethane (C_2_)	2.76	2.73	2.92	3.18		0.0739
Propane (C_3_)	1.42	1.27	1.70	1.01		0.0223
iso-Butane (C_4_)	0.24	0.22	0.31	0.28		0.0027
n-Butane (C_4_)	0.18	0.18	0.24	0.24		0.0042
iso-Pentane (C_5_)	0.03	0.04	0.05	0.13		
n-Pentane (C_5_)	0.02	0.02	0.02	0.08		
N_2_	8.65	9.00	10.51	0.25	1.14	16.92*
CO_2_	5.49	5.60	1.43	1.41	97.87	72.10
H_2_S	0.06	0.21	0.07		0.28	1.03
Totals	100.00	100.00	100.00	99.04		100.05
Sum (C_1_-C_5_)	85.81	85.20	87.99	97.38		9.99
C_1_/(C_2_ + C_3_)	19	20	18	22		107.5
Butane iso/n	1.3	1.2	1.3	1.2		0.64
Pentane iso/n	2.1	2.2	2.2	1.6		
δ^13^C_C1_	-44.0	-43.6	-45.4			-25.9
δ^13^C_C2_	-17.3	-20.3	-16.7			-20.2
δ^13^C_C3_	-21.2	-18.5	-23.1			-20.0
T (in °C)	10.2	15.5	9.6	40 to > 100	> 250	181
Comment	Nearby fluid 41.9 °C		Nearby fluid 23.6 °C			

*N_2_ + O_2_ + Ar, ^#^converted.

The proportion of CH_4_ (C_1_) relative to the other gas components N_2_, CO_2,_ and H_2_S is much higher than in other epithermal and/or hydrothermal systems that are known to emit CH_4_ (e.g., Calypso^37^ Milos^38^ or that are directly associated with hydrocarbon seepage (e.g., Guaymas Basin e.g.,^39^). At Karambusel the proportion of hydrocarbons in the gas phase exceeds 85 vol% resembling the compositions of natural gas reservoirs even though non-hydrocarbon constituents (N_2_, CO_2_, H_2_S; Table 1) e.g.,^35^ also have elevated concentrations. The non-hydrocarbon gases may originate from the thermal decomposition of organic matter (N_2_) and/or the thermochemical sulfate reduction processes (CO_2_, H_2_S) e.g.,^40^. The very high CH_4_ content of > 80 vol% is unique to Karambusel compared to other epithermal and/or hydrothermal systems where the proportion of CO_2_ exceeds that of CH_4_. In addition, the proportion of longer chain hydrocarbons such as ethane (C_2_), propane (C_3_), butane (C_4_) and pentane (C_5_) is particularly high, exceeding the range of abiogenic CH_4_ sources but resembling the composition of natural gas or gas condensate systems.

**Fig. 6:**
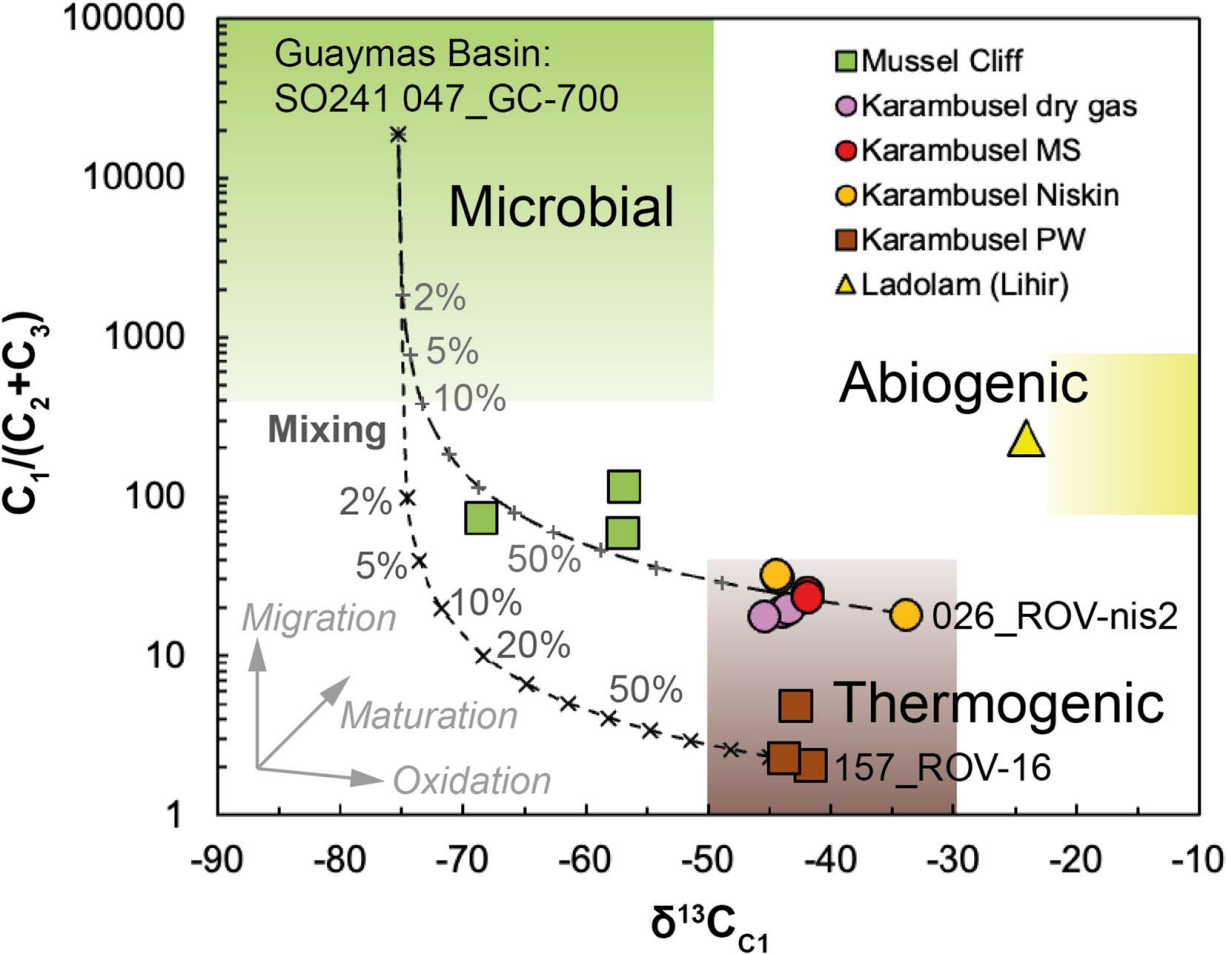
Hydrocarbon gas composition plotted in a modified Bernard diagram (C_1_/(C_2_ + C_3_) vs. δ^13^C_C1_ in ‰ V-PDB)^41^ indicates a thermogenic hydrocarbon source. The data plots along a mixing line (dashed line) of thermogenic and microbially derived methane (10% increments if not labelled otherwise). Endmembers are a headspace sample extracted from a pushcore containing residual CH_4_ hydrates within the Karambusel vent field (SO299 157_ROV-16) and from a *Niskin* bottle collected at a ~ 30 °C warm vent at the southern flank of Karambusel (SO299 026_ROV-nis2), and a seep sample from the Guaymas Basin (SO241 047_GC-700)^39^. Note that for Mussel Cliff only samples from below the sulphate-methane transition zone (i.e., where CH_4_ is least affected by microbial processes) are included.

The C_1_/(C_2_ + C_3_) along with the carbon isotopic composition of CH_4_ (δ^13^C_C1_) can be used to infer the origin of the hydrocarbons. Our new samples from Karambusel plot in the thermogenic field of light hydrocarbons with lower C_1_/(C_2_ + C_3_) in the porewater extracted from pushcores and higher ratios in the captured gas bubbles and vent fluid samples (Fig. 6). One sample collected at the flank of Karambusel at a ~ 30 °C warm vent site (026_ROV-nis2) records heavier carbon isotope ratios than the porewater samples. This difference could be explained by e.g., phase separation of migrating fluids prior to venting, minor admixing of microbial methane to the original fluid, or secondary oxidation of hydrocarbons in the subsurface (Fig. 6). Samples collected at Mussel Cliff, for example, plot along a mixing curve of CH_4_ of microbial (δ^13^C below − 50‰) and thermogenic origin (Fig. 6). Small contributions of methane derived from microbial methanogenesis cannot be excluded as an admixture to the original fluids at Karambusel. However, the high proportion of CH_4_ relative to other gases, and low proportion relative to longer chain hydrocarbons (C_2_ to C_5_), and relatively higher δ^13^C_C1_ at Karambusel all favor thermal maturation of organic-rich sediments over an abiogenic origin. This contrasts with previous observations from natural vents in the Luise harbor (offshore of the Ladolam Au deposit). Here, the CO_2_-dominated geothermal system emits only small amounts of hydrocarbons (≤ 0.71 vol%, Table 1) that are suggested to be of abiogenic origin (Fig. 6)^10^. The thick sedimentary strata of the New Ireland Basin include Miocene volcaniclastic and carbonate rocks (equivalent to the *Lossuk River* beds and the *Lelet* limestone of New Ireland) that may act as potential hydrocarbon source rocks due to their deep burial (> 4 km)^2^. Preliminary n-alkane analyses indicate a mixed input of dominantly land plants and marine algae. The stable carbon isotope composition of C_1_-C_3_ (Fig. S7) is consistent with such a mixed hydrocarbon source^42^ and organic matter degradation induced by geothermal heating^43,44^.

### The endemic Karambusel vent fauna

**Fig. 7:**
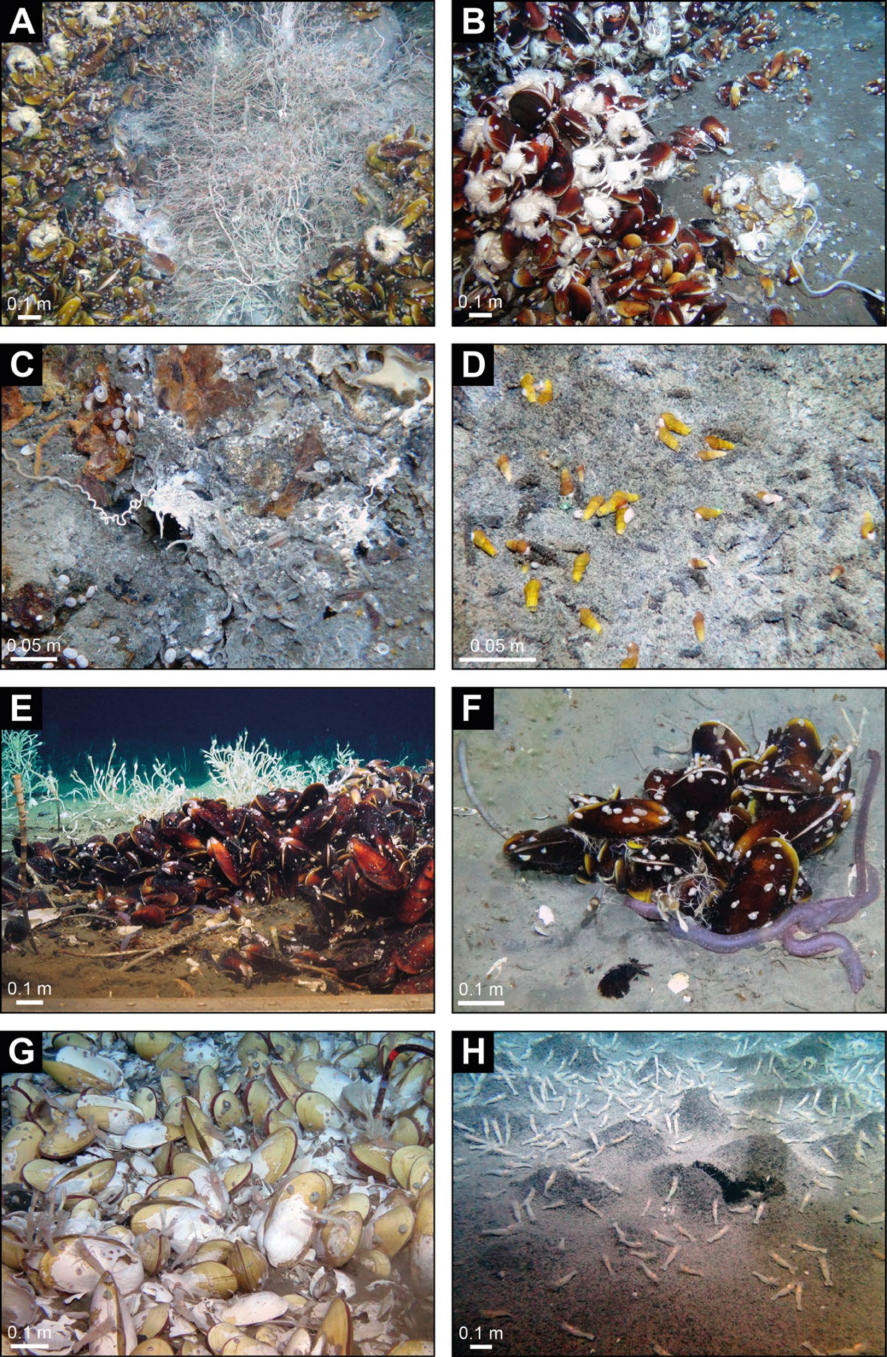
Chemosymbiotic faunal communities of the Karambusel vent field (**A**-**D**), Mussel Cliff (**E**, **F**), and Edison Seamount (**G**, **H**). Karambusel: (**A**) focused, warm vent (~ 29 °C) with dense populations of syboglinids, *Bathymodiolus* mussels and *Shinkaia* crabs, (**B**) *Shinkaia* crabs, Alvinocarididae shrimps, *Bathymodiolus* mussels, gastropods (*Phymorhynchus* and *Paralepetopsis)*, (**C**) Microbial mats on altered volcanic rocks with *Lamellibrachia* curly tube worms, *Paralepetopsis* limpets and *Archinome* hairy worms, (**D**) microbial mats on sediment with *Desbruyeresia* gastropods. Mussel Cliff: (**E**) Dense population of *Bathymodiolus* mussels on carbonate crust; Associated fauna are *Munidopsis* squat lobsters, *Paraescarpia* tube worms, *Paralepetopsis* limpets), ophiuroidea fam. indet., holothuria (*Chiridota*); Note the dispersed *Paraescarpia* tube worms with clusters of *Leucolepas* stalked barnacles in the background; (**F**) Similar faunal assemblage to (**E**) but occurring as an isolated patch around a small fluid outlet; *Sabellidae* feather duster worms are surrounding the *Bathymodiolus* population. Edison Seamount: (**G**) dense cluster of *Calyptogena* gen. inc. vesicomyid clams on volcaniclastic substrate, Alvinocarididae shrimps, *Munidopsis* squat lobsters, *Leucolepas* stalked barnacles and *Bathymargarites* gastropods are associated, (**H**) Close-up of the Spionidae ‘worm carpet’ populated by Alvinocarididae shrimps.

The dominating megafauna 50 to 100 m distant from the active vents (Fig. 1D) are dense mussel beds (*Bathymodiolus* probably of the species *edisonensis* sp. inc.), colonizing the bedrock (Figs. 1D, 2A and 7A-B). Two species of tube worms were observed closest to fluid vents: dense, bush-like and curled *Lamellibrachia columna* sp. inc. and less abundant bamboo-like *Paraescarpia echinospica* sp. inc. (Fig. 7A). Aggregations of *Shinkaia crosnieri* sp. inc. crabs on top of mussel beds occur close to the higher temperature vents (Fig. 7A). Further associated taxa include the stalked barnacles *Leucolepas longa* sp. inc. (Fig. 7A, C), small white bacterial mats (probably *Beggiatoa* gen. inc.) ranging from 1 to 5 m^2^ (Fig. 2D), the gastropod *Phymorhynchus wareni* sp. inc., squat lobster *Munidopsis lauensis* sp. inc., vent shrimp *Alvinocarididae* gen. indet. (INES_2024_0001 and INES_2024_0002) (Fig. 7B), vent endemic fish *Pyrolycus* gen. inc., *Paralepetopsis rosemariae* sp. inc., limpets and the scale worms *Lepidonotopodium* sp. indet. (Fig. 4C). In the periphery of active vents, *L. longa* sp. inc. forms dense aggregations. The gastropod *Desbruyeresia* sp. indet. with its brown, spiny, and spiral shell occurs in areas of whitish sediment (Fig. 7D). Two species of *Alvinocarididae* gen. indet. (INES_2024_0001) and *Alvinocarididae* gen. indet. (INES_2024_0002) were observed grazing on the sediment and the surface of mussel beds near emanating fluids. The presence of *Bathymodiolus* dominated assemblages indicates a well-established or declining successional stage of the hydrothermal system^45^. This assumption is consistent with further associated taxa, such as *Munidopsis lauensis* sp. inc., which also occurs during late successional stages and the absence of *Austinograea* crabs, a pioneering to mid-successional species^45^. The faunal constraints are in line with our observations of a fossil, high temperature and an active, low temperature vent system.

### Hydrothermal venting versus cold seepage South of Lihir

Other regional occurrences of hydrothermal venting and cold seeps may be found at Edison Seamount and Mussel Cliff (Fig. 1B). A widespread diffuse hydrothermal flow has been proposed at Edison Seamount based on faunal observations, whereas Mussel Cliff has previously been interpreted to represent an active cold seep^9,10^.

Edison Seamount is a double-cratered ~ 150 m-high volcanic edifice consisting of unconsolidated volcaniclastic material (Fig. 1B), with no evidence for focused fluid venting, or mineralization. In situ temperature measurements were conducted at spots where fluid flow was suspected but measured temperatures are only slightly above background (max. 4.3 °C, Fig. 1B). The crater rim of Edison Seamount is colonized by extensive vesicomyid clam beds as opposed to the mytilid-dominated fauna at Mussel Cliff. The Edison Seamount beds are dominated by a mix of *Phreagena edisonensis* sp. inc. and *Calyptogena* gen. inc. where hundreds of individuals per square meter are observed (Fig. 7G) cf.^9,46^. Associated fauna are gastropods (*Phymorhynchus wareni* sp. inc.), squat lobster *Munidopsis lauensis* sp. inc. and *Shinkaia crosnieri* sp. inc., shrimps of the genus *Alvinocarididae* gen. indet. (INES_2024_0001) and the stalked barnacle *Leucolepas longa* sp. inc. The barnacles occur in the mussel beds and become the dominant taxon in the periphery. We also observed dense aggregations of the tube worm *Spionidae* gen. indet. These ‘carpets’ appear to cover sediment and vesicomyid communities at their margins and *Alvinocarididae* gen. indet. (INES_2024_0001) seem to graze on their greyish hummocky surface (Fig. 7H). The inside of the aggregations is black and composed of numerous worm tubes containing reddish specimens, similar to those observed in *Ampharetidae* aggregations described from the Guaymas Basin^47^.

Mussel Cliff represents a gentle westward facing and ~ 100 m-high slope rather than a cliff. The slope base strikes N-S and is thought to be structurally (i.e. fault) controlled^1^ (Fig. 1B). Sampling by ROV, video-guided grab and gravity corer recovered massive carbonate concretions and strongly pyritized faunal communities. These sulfide-rich samples are highly porous (~ 30% pore space) to semi-massive and are dominated by pyrite and marcasite almost completely replacing shell fragments and other biogenic matter (Fig. S8). One heatflow station at the foot of Mussel Cliff revealed a high temperature gradient of 3.8 K m^− 1^ and a high heatflow of ~ 3,130 mW m^− 2^ close to a site of fluid discharge. At a few sites, weak outflow of shimmering fluids was observed but in situ temperatures do not exceed 6.7 °C (Fig. 1B) and no gas bubbles were observed. Discounting diagenetic processes in the porewater, our calculated endmember fluids at Mussel Cliff record concentrations of Cl slightly lower than ambient seawater (< 550 mmol L^− 1^; Fig. 5C), but higher concentrations of Ca (~ 58 mmol L^− 1^) and Sr (> 789 mmol L^− 1^) at low Li (< 58 µmol L^− 1^; Fig. 5B) and Si ( < < 1 mmol L^− 1^). The low Li concentrations but high B to Li ratios are consistent with a cold vent/seep system^31^.

The fauna at Mussel Cliff is dominated by mytilid mussels *Bathymodiolus edisonensis* sp. inc., tube worms *Lamellibrachia columna* sp. inc. (small morphotype, filamentous and curved tubes) and *Paraescarpia echinospica* sp. inc. (large morphotype, big bamboo-like tubes), by the stalked barnacle *Leucolepas longa* sp. inc. and holothurians of the genus *Chiridota* sp. indet. (Fig. 7E, F). These communities are most common at the base of Mussel Cliff (Fig. 1B) where they populate scattered small fluid discharge sites (Fig. 7F) or cracks in the carbonate crusts that may act as pathways for migrating fluids. Tube worms *Sabellidae* gen. indet. occasionally form dense aggregations in areas between venting sites (Fig. 7F). A full list of vent endemic taxa at Edison Seamount, Mussel Cliff and the Karambusel vent field can be found in Table 2.

**Table 2:** List of all 23 vent endemic taxa observed at edison seamount, mussel Cliff and the Karambusel vent field and identified by ROV video footage and imagery. Abbreviations after Horton et al.^48^: fam. – family, gen. – genus, inc. – incerta (uncertain identification), indet. – indeterminabilis (taxon is indeterminable beyond a certain taxonomic level), sp. – species.

Phylum/ taxa	Edison seamount	Mussel cliff	Karambusel vent field
Annelida			
*Archinome jasoni* sp. inc.			✓
*Lamellibrachia* c*olumna sp*. inc.		✓	✓
*Lepidonotopodium* gen. inc.			✓
*Paraescarpia echinospica* sp. inc.	✓	✓	✓
Sabellidae gen. indet.		✓	
Spionidae gen. indet.	✓		
Arthropoda			
*Alvinocarididae* gen. indet. (INES_2024_0001)	✓	✓	✓
*Alvinocarididae* gen. indet. (INES_2024_0002)	✓		✓
*Leucolepas longa* sp. inc.	✓	✓	✓
*Munidopsis lauensis* sp. inc.	✓	✓	✓
*Shinkaia crosnieri* sp. inc.	✓	✓	✓
Chordata			
*Pyrolycus* gen. inc.	✓		✓
Cnidaria			
Actinostolidae gen. indet.	✓		
*Maractis* gen. inc.	✓		✓
Echinodermata			
*Chiridota* sp. indet.	✓	✓	
*Ophiuroidea* fam. Indet.			
Mollusca			
*Bathymargarites* gen. inc.	✓		
*Bathymodiolus edisonensis* sp. inc.		✓	✓
*Calyptogena* gen. inc.	✓		
*Paralepetopsis rosemariae* sp. inc.	✓	✓	✓
*Phreagena edisonensis* sp. inc.	✓		
*Phymorhynchus wareni* sp. inc.	✓	✓	✓
*Desbruyeresia* sp. indet.		✓	✓

Edison Seamount is exclusively populated by vesicomyid clams, whereas mytilid mussels dominate Mussel Cliff and the Karambusel vent field. This faunal difference is more likely to result from local environmental factors (e.g., substrate, nutrients), rather than symbiont type alone cf.^49,50^. However, overall, the faunal communities show close similarities between all three sites investigated (Table 2). Only a few of the listed taxa appear to be endemic to only a single habitat. One example is the scale worm *Archinome jasoni* sp. inc. that is endemic to hydrothermal vents^51^. In our study, twenty-three megafauna taxa, including probably two *Calyptogena* clam species, were identified based on ROV imagery. This expands the previously observed number of taxa from 17 at Edison Seamount and Mussel Cliff^8^ to 23, across the three vent and seepage areas.

## Implications

Karambusel is the youngest expression of volcanism in the Lihir island chain and ~ 200 ka younger than the Conical main edifice cf.^1^. However, our new radiometric ages obtained from lavas at Karambusel, and apparent U-Th ages of the mineralization at Conical Seamount (93.4 ± 6.7 ka; Tab. S9) overlap. The high temperature mineralization has Pb isotopic compositions intermediate between Karambusel and Conical Seamount lavas, whereas the low temperature mineralization is isotopically less radiogenic and more similar to the Conical host rock (Fig. 3C). We interpret this to reflect the change in sulfide mineral formation from being dominated by epithermal processes (degassing magma of the Karambusel volcanic event) to leaching of the host rocks by hydrothermal processes (Conical Seamount). We thus conclude that the mineralization at both localities, Karambusel and Conical Seamount, were triggered by the younger magmatic event at Karambusel.

**Fig. 8:**
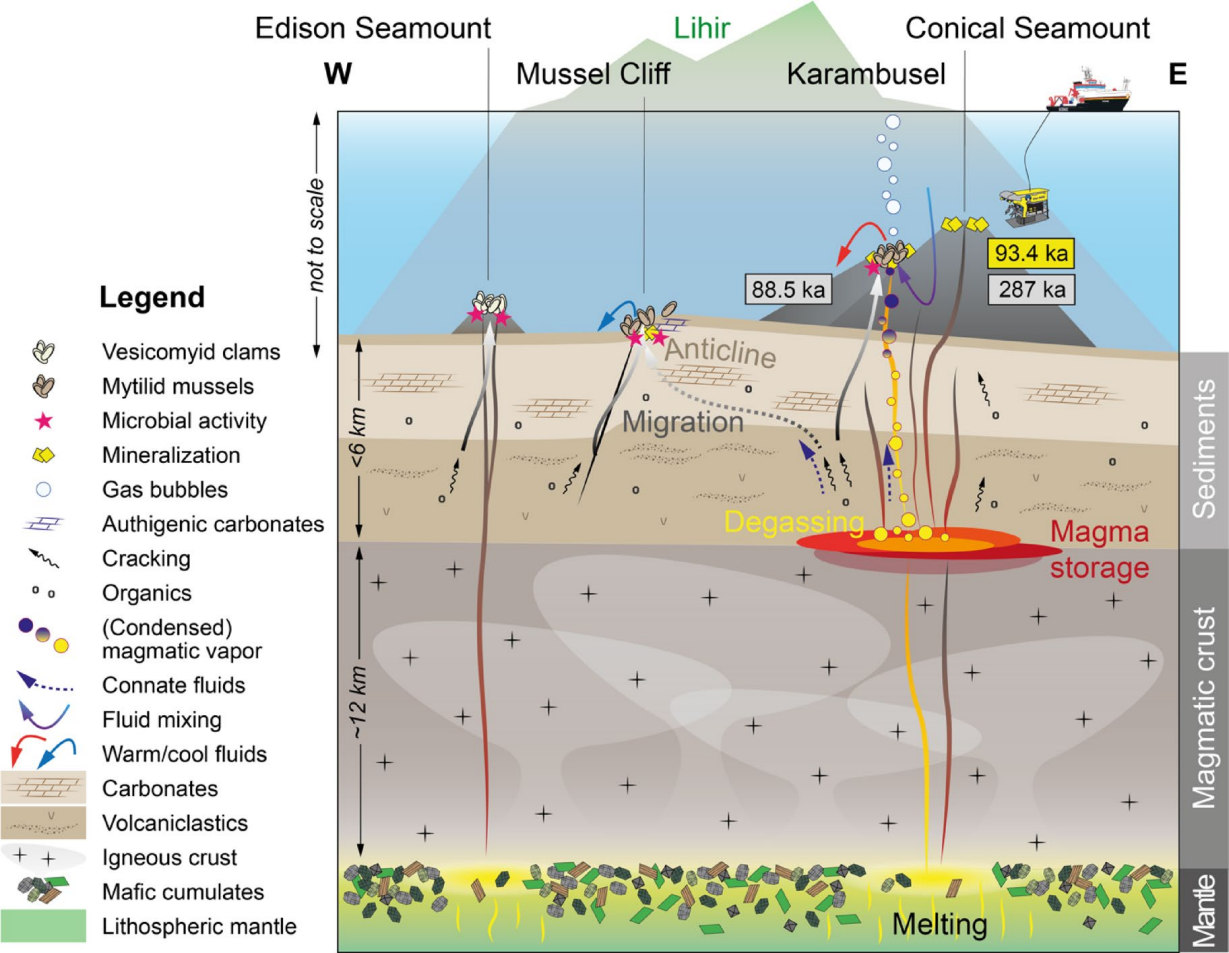
Sketch of the volcanic, venting and seepage systems across Edison Seamount, Mussel Cliff, Karambusel and Conical Seamount (see Fig. 1A for the approximate position of the profile). Volcanic Ar-Ar ages in grey boxes, U-Th ages of sulfides in the yellow box.

This interpretation is consistent with observations from other volcanic plumbing systems where magmatic fluids and gases migrate along the volcanic conduit^52^. We therefore propose a model in which fluids and gases at both localities exsolve from a shallow crustal magmatic reservoir and contribute to the Au-rich mineralization but choose different migrations paths: one along the newly created magmatic feeder system of Karambusel (Fig. 8) and one along the older system leading to the top of Conical Seamount.

Our new eruption and mineralization ages post-date the main epithermal Au stage (600 − 190 ka) at Ladolam^4^. We thus suggest that the mineralization at Ladolam and Conical Seamount, including Karambusel, cannot be part of the same magmatic event as suggested previously^3^. We conclude that the Conical-Karambusel system can be indeed considered as a juvenile analogue to the Luise volcano (Ladolam) as only multi-phase magmatic-hydrothermal ore-forming events have the potential to build an economic deposit^53^.

There is compelling mineralogical and geochemical evidence (e.g., co-precipitation of chalcopyrite, sphalerite, and galena, silicification, Ag-Au-rich mineralization) for a past, high-temperature ore stage linked to a magmatic event, whereas currently, only low-temperature venting is observed. However, the composition of this vent fluid is consistent with a magmatic vapor origin (Fig. 5C, D). Cooling of a super-critical fluid exsolved from a degassing magma leads to phase separation into a brine and a low salinity vapor phase. Such a vapor phase is enriched in Au, As, Sb, Hg, Tl and to a lesser extent Ag and Cu, and condensates to an aqueous liquid during further cooling e.g.,^54,55^ (Fig. 8). For Karambusel, we suggest that sub-critical phase separation (i.e., retrograde boiling) at or close to the seafloor (~ 340 °C) triggered the polymetallic and Au-rich stage of the mineralization. In the active hydrothermal system, boiling and/or mixing with seawater is likely to be confined to the subseafloor, and thus only the most volatile elements (As, Sb, Tl and Hg) will be transported to the seafloor. Cooling of the vapor condensate and mixing with seawater may control the precipitation of the As-Sb-Hg-Tl-rich minerals phases associated with the recent vents (Fig. 8). The mineralization history at Karambusel is thus comparable to that of the Calypso vents offshore New Zealand (Bay of Plenty) where the same suite of elements shows comparable enrichments^56,57^.

Submarine hydrothermal vents hosted in sedimentary basins commonly discharge CH_4_ but their proportion is typically < 10 vol% and CO_2_ dominates the gas phase (~ 50–90 vol%)^37,38,58^. The extremely high proportion of CH_4_ (> 80 vol%) in the dry gas and the concomitant emission along with warm fluids in the deep sea (> 1,000 mbsl) is a unique feature of the Karambusel vent field. We have shown that the CH_4_ at Karambusel is mainly of thermogenic origin but the reduction of (volcanically derived) CO_2_ could further contribute to the high proportion of CH_4_ through the generalized redox-dependent reaction:

$${\text{CO}}_{{\text{2}}} + {\text{ 4 H}}_{{\text{2}}} { \leftrightharpoons }{\text{CH}}_{{\text{4}}} + {\text{2H}}_{{\text{2}}} {\text{O}}^{{{\text{e}}.{\text{g}}.,\;{\text{59}},{\text{60}}}}$$

The volatility ratios of both gas species diverge with decreasing temperature with CH_4_ preferentially partitioning into the gas phase^59^. However, at the fluid temperature measured at Karambusel (~ 50 °C), CH_4_ would be enriched ~ 25% over CO_2_^59^ – too low to significantly shift their relative proportions. In addition, the reaction kinetics at the low temperatures observed at Karambusel are too slow to allow for a sufficient conversion of CO_2_ to CH_4_ assuming residence times of less than 1 ka^61^. Thus, we interpret the large amount of CH_4_ to be a primary feature resulting from the degradation of organic-rich sediments in the basin through magmatic heat^37,44^ (Fig. 8). This contrasts with the active geothermal system at the Ladolam Au deposit, where CO_2_ dominates (97.8 vol%) and CH_4_ is suggested to be of abiogenic origin (high C_1_/(C_2_ + C_3_) and only mildly negative δ^13^C_1_ values)^10^.

The hydrocarbons migrate upwards but require pathways such as volcanic conduits^62^ or faults e.g.,^31^. In the case of Mussel Cliff, the seepage is clearly aligned along a N-S trending fault that cuts off a NW-SE oriented anticline (Figs. 1B and 8). This anticline was seismically imaged during SO94^1^ and may act as a structural trap for ascending fluids and gases. The bathymetric high SE of Mussel Cliff is the surface expression of this structure (Fig. 1A). Faults are also visible at the seafloor around Conical Seamount (Fig. 1A), but it remains inconclusive whether these faults are controlled by crustal structures or are a result of compaction of the sedimentary package through loading by the volcanic edifice. It is, however, less ambiguous that magmatic-hydrothermal fluids and hydrocarbon-rich gases use the same migration paths within the volcano, as evident from their interspersed emission sites within the Karambusel vent field (Fig. 8). This reveals the unique nature of Karambusel and contrasts with other sedimentary (rift) basins such as the Guaymas Basin^39,44^ or the Okinawa Trough^58^ where the seepage of hydrocarbons is spatially separated by at least hundreds of meters to kilometers from the hydrothermal vent sites.

Figure 8 summarizes working hypotheses regarding the source of the fluids. The current discharge is at least partly a product of (inferred) subsurface boiling and possible gas exsolution via hydrocarbon immiscibility. Such phase separation is a response to magmatic activity but may also be occurring in deep formation waters (‘connate fluids’ in Fig. 8) within the sedimentary basin. Generation of hydrocarbons in basins through the burial and thermal maturation of organic-rich sediments is a common process e.g.,^63^. In the New Ireland Basin, residual formation waters may have separated into a water-rich and a hydrocarbon-rich fluid containing both low and high molecular weight hydrocarbons. Although the origin of these hydrocarbons is uncertain, subseafloor structures have been identified that could potentially host hydrocarbon reservoirs (‘anticline’ in Fig. 8). Our new fluid trace element data plot within the ‘sedimentary influenced’ and ‘magmatic vapor’ fields (Fig. 5), highlighting the potential overlap of fluid, gas, and metal sources. The enrichment of As, Sb, Hg and Tl (the so-called epithermal suite of elements^64^ is thought to reflect enrichment in the source rocks, including sediments in the basin and alkaline volcanic rocks, low temperatures of mobilization and transport of these elements, strong partitioning into the vapor-phase fluids and possible magmatic contributions, all of which are possible. The present data do not support a single coherent narrative of ore formation and point instead to a hybrid system intermediate in character between Carlin-like epithermal style mineralization cf.^30^ and hydrocarbon accumulation and transport within the sedimentary basin.

The western Pacific is known for its high hydrothermal vent endemicity and species radiation within each region, coupled with limited faunal connectivity between these regions^65,66^. In this context, our discovery of the first hydrothermal vent field in the TLTF island chain has important implications to understand the distribution of vent specific fauna at a system level. We identified several taxa at Karambusel not previously described from the Mussel Cliff and Edison Seamount nearby. Detailed taxonomic, microbiologial, and genomic studies are required to fully explore the diversity of vent fauna along the TLTF island chain and its connectivity to other vent systems of the western Pacific.

## Conclusions

The Karambusel vent field at the western flank of Conical Seamount is the first discovery of a hydrothermally active deep-sea vent system along the TLTF island chain. The vent field is hosted by a lithologically distinct eruptive center on the western flank of Conical Seamount emplaced ~ 200 ka after the main edifice but similarities in radiometric ages and Pb isotopic composition indicate that the Au-rich epithermal mineralization at both the top of Conical Seamount and Karambusel formed during the same event. The fossil mineralization is subsequently overprinted by a low temperature As-Sb-Hg-Tl-rich stage that is linked to the currently active vent system. A unique feature of the Karambusel vent system is the concomitant but spatially separated discharge of warm (< 51 °C) hydrothermal fluids and hydrocarbon-rich gas at cooler (generally < 20 °C) fluid vents. The proportion of CH_4_ in the dry gas phase at Karambusel exceeds that of any other known hydrothermal system globally. Methane and other hydrocarbons are likely thermogenic in origin with rapid degradation of organic matter linked to the high heat gradient close to the magmatic-hydrothermal system. We argue that the peculiar nature of combined venting and seepage at the Karambusel vent field creates a unique ecological niche that supports a highly endemic chemosymbiotic fauna.

We note here that the survival of this highly endemic fauna could be increasingly threatened by growing economic interests and activities including the disposal of mine tailings (active mining at Ladolam) as well as the potential future exploitation of mineral and hydrocarbon resources in the deep sea (active offshore exploration licences). Urgent and intensified efforts in scientific research, marine spatial planning and environmental protection are critical to mitigate these threats.

## Methods

### Seafloor observations and sampling

Research cruise SO299 DYNAMET with the German RV Sonne took place between 6th June and 29th July 2023 (Townsville to Singapore). The ship is equipped with a Kongsberg Maritime SIMRAD EM122 multibeam echosounder system that operates at 12 kHz. A sound velocity profile was obtained at the beginning of the cruise NW of Lihir Island using a Valeport sound velocity probe. The heatflow was determined using the ‘hard ground heat flow probe’ of the BGR, Hannover. It features a 2.2 m-long sensor rod that contains 7 thermistors (temperature related resistors) with a spacing of 28 cm. The undisturbed temperature gradient in the seafloor was determined only after the frictional heat component caused by the penetration of the rod into the sediments had decayed (10 min). Thereafter, a constant electrical current was sent through a heating wire (about 4 m long) in the sensor rod for 10 min for the determination of the in-situ thermal conductivity. For visual observations and in situ measurements and sampling of the seafloor, the Remotely Operated Vehicle (ROV) *Kiel 6000* of the GEOMAR in Kiel was deployed. The vehicle is a research-optimized work class ROV (type QUEST) manufactured by Schilling Robotics LLC, Davies, USA. The ROV is equipped with two manipulators, a Seabird 49 conductivity-temperature-depth (CTD) probe and several SD and HDTV cameras. A mobile Sonardyne ultra-short baseline (USBL) system was used for underwater navigation and observations were recorded using the ocean floor observation protocol (OFOP) software. During seven dives to Conical Seamount/Karambusel, Mussel Cliff, and Edison Seamount, sampling was carried out using the ROV’s manipulators, 0.3 m-long push cores, two trace metal clean 5 L PVC and Teflon-coated *Niskin* bottles, two 750 mL titanium *Major Samplers* equipped with a nozzle (provided by GNS Science, New Zealand) and stainless-steel gas-tight funnel samplers. A temperature probe was fitted to the suction sampler to allow for in situ temperature measurements.

### Sample preparation and geochemical analyses of volcanic rocks

Rock samples were described on board and cut for thin section preparation and geochemical analyses. Samples were shipped to Germany by container. Thin sections were prepared by the Thin Section Lab (TSL) in Toul, France and Dettmar Dissection Technology (DDT) in Bochum, Germany. The petrography was determined at GEOMAR, Kiel, Germany, using a camera-equipped Zeiss Axio.Imager Z2m. Samples for geochemical analyses were washed in deionized water, crushed using a steel jaw crusher, and sieved. Rock chips of the 1–2 mm-fraction were hand-picked and powdered using an agate mortar and agate planetary mill at GEOMAR, Kiel, Germany.

Major element analyses (SiO_2_, TiO_2_, Al_2_O_3_, Fe_2_O_3_^total^, MnO, MgO, CaO, Na_2_O, K_2_O, P_2_O_5_) of bulk volcanic rock powders were carried out by X-ray fluorescence on a Spectro XEPOS PLUS at the GeoZentrum Nordbayern in Erlangen, Germany. The results of 15 representative samples from Karambusel and two basaltic reference materials are presented in Tab. S1.

The Pb isotope compositions of rock powders were determined at the Institute for Mineralogy, University of Münster. All sample material was leached in 6 N HCl for about 2 h in an ultrasonic bath, followed by leaching in 6 N HCl for about 2 h at 100 °C to minimize the effects of seawater alteration^67^. Sample powders were dissolved in concentrated HF: HNO_3_ (4:1) for 3 days at 140 °C. After drying the samples at 120 °C they were re-dissolved in about 5 ml of 6 N HCl at 120 °C for one hour and dried down thereafter. The separation and purification scheme for Pb is provided in detail in Sani et al.^68^. Lead radiogenic isotope ratios were determined on a Thermo Scientific NEPTUNE Plus MC-ICP-MS at the University of Münster. For the Tl-doped Pb isotope analyses via MC-ICP-MS, accuracy and precision was verified using BCR-2 and BHVO-2 (Tab. S2). Our results for the BCR-2 and BHVO-2 reference materials agree well with reported values^69^. A careful evaluation of our data relative to those reported by Kamenov et al.^12^ (e.g., normalizing to the same NBS-981 values) confirm the small but significant difference in Pb isotopes between samples from Conical Seamount and Karambusel (Fig. 3C). All data are given in Tab. S2.

### ^40^Ar-^39^Ar age dating of mineral separates

Phlogopite and amphibole crystals were separated from either the 150–215 μm or the 215–315 μm fractions using a Frantz isodynamic magnetic separator and were hand-picked under the binocular stereomicroscope. The disc containing the hornblende samples was irradiated for 20 min alongside the Fish Canyon standard^70^ for which an age of 28.294 Ma (± 0.13%) was used^71^. The discs were Cd-shielded (to minimize undesirable nuclear interference reactions) and irradiated in the Oregon State University nuclear reactor (USA) in the central position. The mean J-values computed from standard grains within the small pits yielded values of 0.0000955 (± 0.19%). Mass discrimination was monitored regularly through the analysis using an automated air pipette and provided mean values of 0.993725 (± 0.04%) and 0.993933 (± 0.03%) per dalton (atomic mass unit) relative to an air ratio of 298.56 ± 0.31^71^. The correction factors for interfering isotopes were (^39^Ar/^37^Ar) Ca = 6.95 × 10^− 4^ (± 1.3%), (^36^Ar/^37^Ar) Ca = 2.65 × 10^− 4^ (± 0.84%) and (^40^Ar/^39^Ar) K = 7.30 × 10^− 4^ (± 12.4%^72^). The ^40^Ar/^39^Ar analyses were performed at the Western Australian Argon Isotope Facility at Curtin University, Perth, Australia. Two populations of ~ 30 mg of amphibole each and one population of ~ 30 mg of phlogopite were step-heated using a continuous 100 W PhotonMachine CO_2_ (IR, 10.6 μm) laser fired on the crystals for 60 s. Each of the standard crystals was fused in a single step.

The gas was purified in an extra low-volume stainless steel extraction line of 240 cm^3^ and using two SAES AP10 and one GP50 getter. Argon isotopes were measured in static mode using a low volume (600 cm^3^ ARGUS VI mass spectrometer from Thermo Fisher^74^ set with a permanent resolution of ~ 200. Measurements were carried out in multi-collection mode using four faradays to measure mass 40 to 37 and a low background compact discrete dynode ion counter to measure mass 36. We measured the relative abundance of each mass simultaneously using ten cycles of peak-hopping and 16 s of integration time for each mass. Detectors were calibrated to each other electronically and using air shot beam signals. The raw data were processed using the ArArCALC software^75^ and the ages have been calculated using the decay constants recommended by Renne et al.^63^. Blanks were monitored every three to four steps. All parameters and relative abundance values are provided as supplementary files F1-F3 and have been corrected for blank, mass discrimination and radioactive decay. Individual errors are given at the 1σ level.

Our criteria for the determination of plateau are as follows: plateaus must include at least 70% of ^39^Ar. The plateau should be distributed over a minimum of three consecutive steps agreeing at 95% confidence level and satisfying a probability of fit (P) of at least 0.05. Plateau ages are given at the 2σ level and are calculated using the mean of all the plateau steps, each weighted by the inverse variance of their individual analytical error (Fig. S3). Uncertainties include all sources of uncertainties where the decay constant and age of the standard uncertainties are negligible compared to the analytical uncertainties.

### Petrography and bulk rock chemistry of mineralized samples

Representative and large volume (i.e. several hundred grams each) splits of altered and/or mineralized rock samples were washed with deionized water, dried, and subsequently crushed and milled to a grainsize of ~ 50 μm. The rock powders were analyzed for their major, trace, and precious metal concentration by a variety of techniques at Activation Laboratories Ltd. (Actlabs) in Ancaster, Canada. A table with methods applied and analytical results relevant to this contribution is presented as Tab. S4.

Polished thin and thick sections of the mineralized samples were petrographically examined with transmitted and reflected light microscopy to identify the different mineral phases, textures, and paragenetic relations. Energy-dispersive X-ray spectrometry was performed at the GeoZentrum Nordbayern, Erlangen, Germany, by a Hitachi TM4000 tabletop scanning electron microscope in order to identify unknown mineral phases and provide semi-quantitative element concentrations for sulfide minerals.

### Fluid and gas chemistry

Two *Niskin* bottles were mounted in a horizontal configuration on the front of the portside drawer of the ROV. The *Niskin* samplers were standard 5 L PVC, Teflon-coated samplers triggered by an outer spring to avoid metal contamination of the sampled fluid. The bottles were closed via color-coded ropes using the ROV arm. The 750 mL titanium *Major Sampler* was used to sample focused fluids directly at the vent output via a titanium nozzle. Prior to triggering the *Major Sampler* via the ROV arm, the nozzle was flushed for at least 3 min in the hydrothermal fluid. The *Major Sampler* was left in the fluid for 3–5 min to allow the sample volume to decrease due to cooling without drawing in surrounding seawater. Fluid samples were always taken after a temperature measurement with a calibrated temperature sensor, and always before sediment samples or rock samples were taken to avoid contamination. Stainless steel gas-tight samplers with a funnel attached to the inlet were deployed directly over vent outlets, i.e., with visible gas bubbling, using the ROV arm. The gas-tight sampler was held in place until filled, which was visible by the formation of gas hydrates in the funnel. After recovery of the ROV, the *Niskin* Bottles and *Major Samplers* were transferred into the lab for sample processing whereas the gas samplers were directly stored for shipment.

An acid cleaned sample tube was used to retrieve the fluid from the *Niskin* bottles and *Major Samplers*. The tube was initially flushed with 100–200 mL of the collected fluid. This fluid was used for pH measurement (NBS scale) with a calibrated handheld multimeter. Afterwards the fluid sample was retrieved using the flushed tubes. Unfiltered fluids were directly transferred into 100 mL glass bottles for CH_4_ analysis from the *Niskin* bottles and into 20 mL glass vials from the *Major Samplers* and 20 mL polypropylene vials for total alkalinity analysis. A 250 mL aliquot of the unfiltered sample was transferred to a pre-cleaned LDPE bottle, which was used to fill a pre-cleaned syringe for filtering the sample through 0.2 μm Satorious RC filters. New filters were used for each fluid. Filtered water was sampled for total alkalinity analysis (20 mL polypropylene vials), major cations (3 mL) and anions (20 mL). The rest of the sample fluid was transferred (unfiltered) into a 250 mL pre-cleaned LDPE bottle and kept as a library sample. Filter and acid blanks were taken for quality control and assurance of the implemented method. Sample tubing was cleaned in between ROV dives with 0.1 M q-HCl and Milli-Q water. As for sample processing, 10 mL of each sample for CH_4_ analysis was removed with a syringe and immediately replaced by helium with a needle stuck trough the butyl septum. The glass vials were then shaken for 20–30 s and afterwards 0.5 mL of NaOH solution was added. The puncture points in the septum were sealed with silicone and the vials were then placed in a cardboard box at + 4 °C in the dark. After the silicone had dried the vials were turned upside down. Depending on whether enough fluid was available, duplicates were taken. Fluid samples were stored and then transported under climate-controlled conditions (+ 4 °C) via air freight and courier from Singapore to Kiel, Germany.

Fluid samples were analyzed at GEOMAR, Kiel, Germany following standard techniques. Chloride concentrations of fluids were determined by ion-chromatography (Methrom ECO IC). Fluid samples of 200 µL were diluted (1:50) with NaCO_3_/NaHCO_3_-eluent before injecting the sample to the ion chromatograph. The chromatographic separation of chloride from other anions, i.e., bromide and sulfate, was conducted in a ‘Metrosep A Supp 5–150/4.0’ column. The accuracy of the concentration measurements detected by an integrated conductivity cell is about 2% RSD. Inductive Coupled Plasma-Optical Emission spectrometry (ICP-OES) was used to analyze the major elements, i.e., Li, Na, Mg, Ca, Sr, B, and Si on an Agilent 5800 ICP-OES. A 100 µL aliquot of the acidified fluid sample was diluted in pure water (1:50) and spiked with a Y-standard before injection into the argon plasma. Three replicates were measured for each fluid sample. The major elements were determined by a polychromator detecting the emission spectra from 167 to 785 nm wavelengths. Analytical results of samples and 31 measurements of the IAPSO Standard Seawater are presented in Tab. S5. A range of 0.7–1.5% RSD (with the exception of Si: 5.6%) was calculated for the accuracy of the measurements.

The gas-tight funnel samplers consist of 344 bar-pressure-rated and polished stainless-steel vessels, which are connected to plastic funnels by ball valves (Swagelok, 316 L). Gas bubble streams were collected at depth until the inner volume of seawater (300 mL) was replaced by gas. Closed samplers were stored at room temperatures until further processing, i.e., subsampling of gas into headspace vials by using a high-vacuum system (< 10^− 4^ mbar). Headspace vials made of glass crimped with rubber septum and aluminum cap were filled with sampled gas at 1050 mbar for subsequent chemical analyses at the GEOMAR, Kiel. These samples were analyzed using a Shimadzu 2014 gas chromatograph equipped with FID/TCD detectors for gas composition analysis and Thermo Fisher MAT253 continuous flow isotope ratio mass spectrometry for δ^13^C-determination (Table 2 and S6).

## Supplementary Information

Below is the link to the electronic supplementary material.

Supplementary Material 1

Supplementary Material 2

Supplementary Material 3

Supplementary Material 4

## Data Availability

All data generated or analysed during this study are included in this published article and its supplementary information files except for digital elevation model data. Bathymetric data of RV Sonne expedition SO299 are available from PANGAEA under DOI: 10.1594/PANGAEA.968523 and those of expeditions SO94, SO133 and SO166 are available from the authors upon reasonable request. ASTER Global Digital Elevation Model V003 data are available from the NASA EOSDIS Land Processes Distributed Active Archive Center under DOI: 10.5067/ASTER/ASTGTM.003.
