# Supplementary material for: Coupled hydrothermal venting and hydrocarbon seepage discovered at Conical Seamount, Papua New Guinea
Source: Sci Rep. 2025 Sep 19;15:32389. doi: 10.1038/s41598-025-17192-x (PMC12449473; doi:10.1038/s41598-025-17192-x)
Supplement: Supplementary file 1 — Supplementary Material 1 [file 41598_2025_17192_MOESM1_ESM.pdf]

Supplementary Information

Table S1: Major element composition of volcanic rock samples from Karambusel and two basaltic reference materials.

IGSN	Initial ID	Lon	Lat.	WD	SiO_2	TiO_2	Al_2O_3	$Fe_2O_3^T$	MnO	MgO	CaO	Na_2O	K2O	P_2O_5	LOI	Total
		°E	°S	mbsl	wt.%	wt.%	wt.%	wt.%	wt.%	wt.%	wt.%	wt.%	wt.%	wt.%	wt.%	wt.%
KIEL0256GRAB201	026_ROV_01	152.6523	3.3185	1594.6	47.31	0.75	15.78	10.35	0.22	5.91	11.42	3.41	3.14	0.56	0.82	99.66
KIEL0256GRAC201	026_ROV_02	152.6525	3.3180	1561.7	47.07	0.70	16.32	10.40	0.22	5.29	10.69	3.84	3.36	0.59	1.19	99.67
KIEL0256GRAD201	026_ROV_03	152.6518	3.3162	1471.6	47.57	0.70	15.82	10.49	0.22	5.89	11.44	3.44	3.19	0.58	0.33	99.67
KIEL0256GRAE201	026_ROV_04	152.6513	3.3157	1453.2	47.58	0.71	16.99	9.60	0.19	5.09	10.63	3.16	3.13	0.61	2.01	99.69
KIEL0256GRAF201	026_ROV_05	152.6512	3.3155	1439.8	46.86	0.72	16.76	10.44	0.21	5.07	11.08	3.13	3.18	0.67	1.56	99.66
KIEL0256GRKG201	081_ROV_04	152.6458	3.3114	1618.7	47.51	0.77	16.02	10.85	0.21	5.47	11.06	3.47	3.51	0.57	0.23	99.66
KIEL0256GRKH201	081_ROV_05	152.6473	3.3114	1593.9	47.30	0.70	16.44	10.42	0.21	5.17	10.64	3.61	3.46	0.62	1.07	99.65
KIEL0256GRKM201	081_ROV_07	152.6504	3.3114	1430.7	47.91	0.68	17.30	10.01	0.24	4.80	9.97	3.80	2.96	0.62	1.35	99.64
KIEL0256GRRX201	157_ROV_02	152.6472	3.3160	1614.6	47.09	0.71	16.57	10.58	0.21	5.40	10.72	3.33	3.20	0.63	1.22	99.66
KIEL0256GRRY201	157_ROV_03	152.6487	3.3153	1516.3	48.29	0.74	16.99	10.94	0.22	5.45	11.21	3.54	3.45	0.65	-1.84	101.48
KIEL0256GRRZ201	157_ROV_04	152.6493	3.3150	1463.5	46.40	0.72	16.47	10.85	0.22	5.49	11.25	3.39	3.33	0.64	0.90	99.65
KIEL0256GRR5201	157_ROV_08	152.6516	3.3139	1330.2	51.37	0.65	18.01	5.61	0.11	4.28	9.42	2.77	3.68	0.57	3.19	99.65
KIEL0256GRSE201	157_ROV_17	152.6521	3.3137	1341.5	45.87	0.76	15.98	10.82	0.21	5.38	11.34	3.82	3.46	0.59	1.43	99.65
KIEL0256GRSF201	157_ROV_18	152.6510	3.3132	1344.6	49.94	0.75	17.57	5.65	0.13	4.95	11.02	3.21	2.76	0.68	3.00	99.66
KIEL0256GRSG201	157_ROV_19	152.6517	3.3129	1354.3	46.47	0.75	16.15	10.77	0.22	5.59	10.89	3.71	3.30	0.59	1.22	99.66
**Reference material**	**Analyses, references**				**SiO_2**	**TiO_2**	**Al_2O_3**	**$Fe_2O_3^T$**	**MnO**	**MgO**	**CaO**	**Na_2O**	**K2O**	**P_2O_5**	**LOI**	**Total**
BR	This study, n = 2				38.64	2.60	10.42	12.54	0.20	13.01	13.53	3.31	1.35	1.12	2.81	99.51
	Govindaraju, 1995				38.20	2.60	10.20	12.88	0.20	13.28	13.80	3.05	1.40	1.04	3.00	99.65
BE-N	This study, n= 1				38.94	2.64	10.25	12.60	0.21	12.56	14.00	3.30	1.40	1.16	2.44	99.50
	Govindaraju, 1995				38.20	2.61	10.07	12.84	0.20	13.15	13.87	3.18	1.39	1.05	2.45	99.01

IGSN – International Generic Sample Number, LOI – Loss On Ignition

Govindaraju, K. 1995 working values with confidence limits for twenty-six CRPD, ANRT and IW-GIT geostandards. Geostandards Newsletter 19, 1–32 (1995).

Table S2: Pb isotopic composition of volcanic rock samples from Karambusel and two reference materials.

IGSN	Initial ID	$^{206}Pb/^{204}Pb$	2 S.E.	$^{207}Pb/^{204}Pb$	2 S.E.	$^{208}Pb/^{204}Pb$	2 S.E.
KIEL0256GRAB201	026_ROV_01	18.7434	0.0022	15.5528	0.0027	38.3763	0.0090
KIEL0256GRAD201	026_ROV_03	18.7353	0.0018	15.5432	0.0021	38.3460	0.0069
KIEL0256GRAE201	026_ROV_04	18.7804	0.0018	15.5515	0.0022	38.4039	0.0072
KIEL0256GRKG201	081_ROV_04	18.7889	0.0014	15.5561	0.0016	38.4227	0.0054
KIEL0256GRKH201	081_ROV_05	18.7702	0.0022	15.5433	0.0027	38.3704	0.0092
KIEL0256GRKM201	081_ROV_07	18.7730	0.0009	15.5464	0.0009	38.3807	0.0030
KIEL0256GRRZ201	157_ROV_04	18.7762	0.0014	15.5464	0.0016	38.3868	0.0051
KIEL0256GRR5201	157_ROV_08	18.7833	0.0021	15.5594	0.0026	38.4288	0.0086
KIEL0256GRSF201	157_ROV_18	18.7820	0.0010	15.5582	0.0011	38.4222	0.0036
**Reference material**		$^{206}Pb/^{204}Pb$	2 S.E.	$^{207}Pb/^{204}Pb$	2 S.E.	$^{208}Pb/^{204}Pb$	2 S.E.
BCR-2		18.7999	0.0006	15.6237	0.0006	38.8174	0.0018
BHVO-2		18.6682	0.0027	15.5444	0.0022	38.2754	0.0053

IGSN – International Generic Sample Number

Figure S3: Ar age plateaux of phlogopite (‚biotite‘) and amphibole (‚hornblende‘).

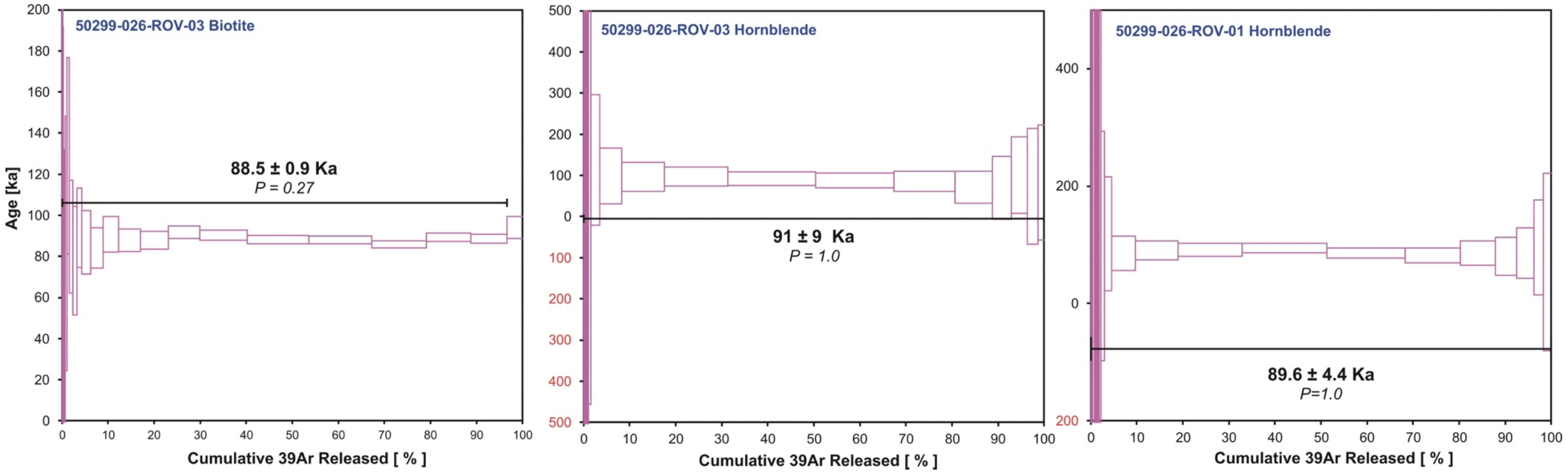

Table S4: Bulk geochemical analyses of altered and/or mineralized rock samples from Karambusel.

IGSN	Initial ID	Lon.	Lat.	WD	Element	Si	Fe	S	Cu	Au	Ag	As	As	Sb	Sb	Hg	Hg	Tl
		152°	3°		Unit	wt.%	wt.%	wt.%	$\mu g\ g^{-1}$	$ng\ g^{-1}$	$\mu g\ g^{-1}$	$\mu g\ g^{-1}$	$\mu g\ g^{-1}$	$\mu g\ g^{-1}$	$\mu g\ g^{-1}$	$ng\ g^{-1}$	$\mu g\ g^{-1}$	$\mu g\ g^{-1}$
		xx.xx‘	xx.xx‘		Tql.	0.01	0.05	0.01	2	2	5	5	0.5	2	0.1	5	1	0.1
		°E	°S		Method	FUS	FUS	CS	FUS-MS	INAA	INAA	FUS-MS	INAA	FUS-MS	INAA	1G	INAA	FUS-MS
KIEL0256GRAB201	026_ROV-01	39.149	19.109	1595		23.5	7.25	0.01	59	bql.	bql.	6	14.4	bql.	2.6	25	bql.	0.2
KIEL0256GRAE201	026_ROV-04	39.077	18.939	1452		22.1	6.37	1.27	77	11	bql.	16	21.1	bql.	2	42	bql.	0.5
KIEL0256GRAH201	026_ROV-07	39.117	18.831	1338		29.7	1.81	8.83	17900	28200*	2410	29400	> 10000	> 5000	5850	> 100000	56	75.6
KIEL0256GRBG201	046_ROV-17	39.579	18.730	1056		22.4	7.24	4.9	134	bql.	bql.	16	3.9	bql.	2.9	186	bql.	1.2
KIEL0256GRKP201	081_ROV-10	39.059	18.831	1330		23.8	4.75	0.83	29	bql.	bql.	41	64.6	4	4.8	66	bql.	0.7
KIEL0256GRKR201	081_ROV-12	39.104	18.833	1335		0.53	42.9	51.8	285	1540	bql.	> 10000	> 10000	766	660	> 100000	238	626
KIEL0256GRKU201	081_ROV-15A	39.115	18.836	1339		34.4	0.59	5.18	101	1120	bql.	65200	> 10000	> 5000	5090	> 100000	507	232
	081_ROV-15B	39.115	18.836	1339		36.4	0.46	0.46	32	195	bql.	5340	4450	116	94.2	> 100000	252	22.8
KIEL0256GRKV201	081_ROV-16	39.120	18.835	1339		33	3.47	0.44	162	897	bql.	4220	3960	206	184	> 100000	117	51.9
KIEL0256GRK2201	081_ROV-21	39.091	18.822	1331		22.5	5.55	2.07	187	bql.	bql.	30	31.5	bql.	2.2	289	bql.	0.4
KIEL0256GRK4201	081_ROV-23	39.099	18.834	1334		3.91	38.9	46.5	922	8500	123	6140	5620	252	219	27900	23	167
KIEL0256GRK5201	081_ROV-24	39.099	18.835	1334		3.1	39.5	48	1910	11300	409	5200	4710	437	375	16700	14	105
KIEL0256GRR5201	157_ROV-08	39.093	18.832	1330		24.4	4.44	1.23	137	bql.	bql.	229	224	48	39	393	bql.	26.2
KIEL0256GRSB201	157_ROV-14	39.112	18.834	1338		40.7	0.79	1.48	329	445	bql.	1150	1050	101	80.8	> 100000	114	108
KIEL0256GRSC201	157_ROV-15	39.088	18.823	1329		21.7	5.01	5.79	161	24	bql.	40	38.9	bql.	1.2	172	bql.	0.2
KIEL0256GRSN201	159_TVG-01	39.121	18.832	1313		31.5	2.58	2.16	184	3470	bql.	1010	945	19	14.6	59900	41	31.5
KIEL0256GRSQ201	159_TVG-03A	39.121	18.832	1313		31.1	6.97	8.25	718	10000	64	5220	4820	101	81.4	> 100000	135	46.7
	159_TVG-03B	39.121	18.832	1313		30.9	5.15	6.16	821	14100	90	2330	2010	133	109	> 100000	226	72.1
	159_TVG-03C	39.121	18.832	1313		31.4	1.94	0.38	98	1600	bql.	64	71.3	3	2.9	87300	67	2.4
KIEL0256GRST201	159_TVG-06	39.121	18.832	1313		30.7	4.64	3.32	108	1530	bql.	770	780	18	16.2	10000	8	30.7
KIEL0256GRSW201	160_TVG-02	39.043	18.876	1375		16.4	16.1	0.18	79	bql.	bql.	146	149	4	3.1	249	bql.	0.3

Tql. – typical quantification limit
Methods: FUS – Na_2O_2 fusion, CS – total sulfur by IR, INAA – Instrumental Neutron Activation Analysis, MS – Mass Spectromtry, 1G – aqua regia Hg cold vapor flow injection technique.
*determined using 60 g fire-assay followed by gravimetry

Please note that a publication including the complete data table is currently in preparation by Falkenberg et al.

Table S5: Major element analyses of fluid samples.

Sample	Type	Lat. (°S)	Lon. (°E)	WD	pH	T	TA	B	Mn	Ca	Fe	Na	Mg	Sr	Si	Ba	Li	K	SO_4	Cl
		3°xx‘	152°xx‘	mbsl		(°C)	meq L^{-1}	mM	µM	mM	µM	mM	mM	µM	mM	nM	µM	mM	mM	mM
Karambusel																				
026_ROV-Nis01	Nis	18.932‘	39.072‘	1440	7.69	9.5	2.45	0.42	bql	10.5	0.10	481	54.1	87.7	0.11	113	26.8	10.2	28.3	555
026_ROV-Nis02	Nis	18.925‘	39.069‘	1431	7.56	29.5	2.47	0.43	bql	10.7	0.10	485	54.5	88.3	0.11	110	27.7	10.3	28.5	553
081_ROV-11nis	Nis	18.833'	39.102‘	1335	7.64		2.70	0.43	bql	10.5	0.05	474	53.2	88.3	0.10	103	26.9	10.2	28.4	553
081_ROV-14	MS	18.836'	39.115‘	1339	5.87	15.5	7.99	1.46	3.72	10.3	1.47	468	49.2	97.4	0.38	153	144	11.4	27.5	536
081_ROV-20nis	Nis	18.822'	39.090‘	1331	6.73	50.4	2.38	0.47	0.33	10.3	bql	466	52.1	86.5	0.12	104	33.2	10.0	28.4	552
157_ROV-06nis	Nis	18.884'	38.983‘	1432	7.33	5.0	2.58	0.42	bql	10.4	0.02	469	53.2	87.3	0.10	96.7	26.7	10.3	28.6	558
157_ROV-10	MS	18.822'	39.091’	1331	5.90	51.1	13.70	2.31	8.54	9.17	3.31	420	41.4	90.0	0.66	201	269	11.9	24.3	476
157_ROV-11nis	Nis	18.822'	39.090‘	1331	6.97	51.1	2.55	0.44	0.02	10.4	0.05	469	53.3	87.6	0.10	100	29.4	10.3	28.6	557
157_ROV-16, BW	BW	18.816'	39.099‘	1341		10.4	14.33	2.27	10.29	9.82	1.03	448	43.8	92.5	0.51	355	266	13.3	25.4	509
157_ROV-16, 0-3 cm	PW	18.816'	39.099‘	1341		10.4	14.75	2.40	12.58	9.65	0.75	443	42.4	93.1	1.00	437	288	14.0	25.2	510
Endmember fluid																				
Best fit						*215*		*8.77*	*37.7*	*5.67*	*14.6*	*231*	*0*	*100*	*2.66*	*411*	*1098*	*19.1*	*11.8*	*191*
Lower bound						*145*		*5.89*	*25.3*	*3.80*	*9.80*	*155*	*0*	*67.3*	*1.78*	*276*	*736*	*12.8*	*7.93*	*128*
Upper bound						*360*		*14.7*	*63.0*	*9.47*	*24.4*	*386*	*0*	*168*	*4.45*	*686*	*1834*	*31.9*	*19.7*	*319*
Conical Seamount																				
046_ROV-20nis	Nis	18.736’	39.555’	1042	7.42		2.36	0.42	bql	10.5	bql	477	53.7	87.6	0.08	94.0	26.7	10.1	28.4	561
Edison Seamount																				
051_ROV-01	Nis	18.825'	34.895‘	1436	7.75	3.4	2.40	0.42	bql	10.5	bql	478	53.8	88.2	0.11	106	26.6	10.2	29.0	561
051_ROV-12nis	Nis	19.035'	34.785‘	1450	7.63	3.9	2.37	0.42	bql	10.5	0.06	478	53.7	88.4	0.11	116	26.6	10.1	28.9	559
051_ROV-17, BW	BW	18.897'	34.902‘	1490			2.37	0.45	bql	11.1	2.33	505	56.7	93.6	0.13	150	28.5	11.1	28.6	554
051_ROV-17, 0-5 cm	PW	18.897'	34.903‘	1490			2.41	0.50	bql	10.1	bql	481	51.8	87.0	0.17	202	31.0	12.0	28.5	554
051_ROV-17, 5-10 cm	PW	18.897'	34.904‘	1490			2.43	0.51	bql	9.93	1.46	480	51.1	86.2	0.19	190	31.5	12.2	28.5	554
051_ROV-17, 10-15 cm	PW	18.897'	34.905‘	1490			2.53	0.51	bql	10.1	bql	485	51.8	87.8	0.20	206	31.7	12.3	28.6	555
051_ROV-17, 15-20 cm	PW	18.897'	34.906‘	1490			2.51	0.51	bql	10.1	0.02	492	52.2	87.3	0.20	191	31.9	12.4	28.6	555
051_ROV-17, 20-25 cm	PW	18.897'	34.907‘	1490			2.55	0.50	bql	9.94	0.01	481	51.1	86.8	0.19	179	31.6	12.3	28.6	555
163_GC, 12 cm	PW	18.967'	34.806‘	1479			2.59	0.50	8.98	10.3	5.15	468	51.1	95.9	0.20	246	25.5	11.3	27.8	560
163_GC, 20 cm	PW	18.967'	34.806‘	1479			2.67	0.50	6.59	10.4	7.99	468	51.5	96.9	0.21	264	24.7	11.2	27.8	559
163_GC, 35 cm	PW	18.967'	34.806‘	1479			2.63	0.52	3.22	10.4	23.7	467	51.0	96.8	0.25	216	25.0	11.4	27.6	558
163_GC, 50 cm	PW	18.967'	34.806‘	1479			2.59	0.53	2.21	10.3	2.77	467	51.2	97.2	0.22	254	27.0	11.3	27.7	562
Mussel Cliff																				
063_ROV-01, BW	PW	19.079'	35.411‘	1593			2.38	0.46	0.29	11.3	1.38	484	53.9	98.9	0.10	455	27.5	10.4	28.2	560
063_ROV-01, 5-10 cm	PW	19.079'	35.411‘	1593			1.70	1.89	0.04	42.5	0.13	411	16.0	575	0.26	48806	38.8	12.2	0.07	537
063_ROV-01, 10-15 cm	PW	19.079'	35.411‘	1593			1.48	1.62	6.65	37.3	0.18	428	23.7	474	0.35	9353	38.7	12.1	6.61	545
093_ROV-01nis	Nis	19.177'	35.403’	1600	7.77	2.9	2.44	0.42	bql	10.4	0.01	469	53.4	87.3	0.11	112	26.6	10.3	29.1	566
093_ROV-03, BW	PW	19.177'	35.403‘	1600			2.87	0.43	bql	10.4	0.12	472	53.9	88.1	0.10	108	26.9	10.4	28.9	565
093_ROV-03, 0-5 cm	PW	19.177'	35.403‘	1600			2.60	0.57	4.26	10.1	49.1	474	51.1	88.5	0.21	237	32.2	12.8		
093_ROV-03, 5-11 cm	PW	19.177'	35.403‘	1600			2.31	0.56	1.46	9.68	9.88	473	49.7	86.3	0.20	279	33.7	13.7	29.0	566
093_ROV-03, 11-15 cm	PW	19.177'	35.403‘	1600			2.64	0.53	0.66	9.66	0.43	474	49.2	90.3	0.22	415	34.2	13.9		
093_ROV-03, 15-22cm	PW	19.177'	35.403‘	1600			2.67	0.55	0.47	10.0	0.39	472	49.6	94.6	0.23	510	33.1	13.2	29.0	565
093_ROV-03, 22-24 cm	PW	19.177'	35.403‘	1600			2.91	0.56	0.38	10.1	0.62	473	49.6	97.5	0.23	533	33.4	13.3		
155_GC, 10 cm	PW	19.201'	35.385‘	1592		3.5	5.67	0.79	0.00	12.1	0.11	465	47.7	124	0.20	673	29.4	11.2	21.8	570
155_GC, 15 cm	PW	19.201'	35.385‘	1592		3.8	7.17	1.05	0.08	14.0	0.14	458	43.9	170	0.21	827	30.2	11.1	16.0	568
155_GC, 20 cm	PW	19.201'	35.385‘	1592		4.0	9.24	1.31	bql	16.6	0.15	447	39.0	237	0.23	1088	31.6	11.0	10.6	545
155_GC, 35 cm	PW	19.201'	35.385‘	1592			10.92	1.90	0.03	21.2	0.64	434	30.5	410	0.33	3238	37.3	11.2	0.35	540
155_GC, 70 cm	PW	19.201'	35.385‘	1592		5.7	5.52	2.72	0.62	38.9	0.02	410	17.6	720	0.51	802	46.8	11.1	0.00	530
155_GC, 95 cm	PW	19.201'	35.385‘	1592		7.0	2.13	3.34	4.24	51.8	0.75	388	10.1	872	0.64	489	52.0	10.9	0.00	518
IAPSO	N=31							0.44		10.5		479	54.0	87.5	0.03	423	27.1	10.4		
RSD (in %)								*0.70*		*1.01*		*0.91*	*0.91*	*1.02*	*5.59*	*1.53*	*0.98*	*1.36*		

↑ WD – water depth, Nis/MS are dissolved trace metal samples, T – *in situ* temperature, TA – Total alkalinity, mM – mmol L^{-1}, µM – µmol L^{-1}, nM – nmol L^{-1}, bql – below quantification limit, BW – bottom water, PW – pore water, Nis – *Niskin* bottle, MS – *Major Sampler*, PC – push core, GC – gravity core

Table S6: Low molecular weight (methane – C_1, ethane – C_2, propane – C_3) hydrocarbon analyses and stable carbon isotope composition in per mil relative to Vienna-Pee Dee Belemenite (V-PDB) of headspace samples.

Sample	type	locality	Lon.	Lat.	WD	CH_4	C_2H_6	C_3H_8	$C_1/(C_2+C_3)$	$\delta^{13}C_{C1}$	$\delta^{13}C_{C2}$	$\delta^{13}C_{C3}$
						Methane	Ethane	Propane		Methane	Ethane	Propane
			°E	°S	mbsl	ppmV	ppmV	ppmV		‰	‰	‰
026_ROV-Nis02	Nis	Karambusel	152°39.069'	3°18.925'	1431	3214	142	31.8	18.5	-34.00	-18.52	-15.48
081_ROV-20nis	Nis	Karambusel	152°39.090'	3°18.822'	1331	52201	1371	296	31.3	-44.39	-20.25	-23.78
157_ROV-11nis	Nis	Karambusel	152°39.090'	3°18.822'	1331	19417	487	106	32.7	-44.53	-17.83	-16.79
157_ROV-16, BW	PC	Karambusel	152°39.099'	3°18.816'	1431	7590	1677	1943	2.10	-41.74	-19.43	-22.52
157_ROV-16, 3-6 cm	PC	Karambusel	152°39.099'	3°18.816'	1431	3435	701	781	2.32	-43.87	-19.41	-21.58
157_ROV-16, hydrate residue	PC	Karambusel	152°39.099'	3°18.816'	1431	5841	437	733	4.99	-42.95	-16.69	-19.00
081_ROV-14	MS	Karambusel	152°39.115'	3°18.836'	1339	22333	746	131	25.5	-42.00	-19.70	-17.89
157_ROV-10	MS	Karambusel	152°39.091'	3°18.822'	1331	19911	695	133	24.1	-41.94	-17.23	-17.92
155-GC, 5 cm	GC	Mussel Cliff	152° 35,385'	3°19,201'	1595	19.9				-34.62		
155-GC, 10 cm	GC	Mussel Cliff	152°35.385'	3°19.201'	1595	152	2.09		72.4	-43.21		
155-GC, 15 cm	GC	Mussel Cliff	152°35.385'	3°19.201'	1595	102	0.930		110	-32.43		
155-GC, 20 cm	GC	Mussel Cliff	152°35.385'	3°19.201'	1595	371	3.51		106	-41.27		
155-GC, 35 cm	GC	Mussel Cliff	152°35.385'	3°19.201'	1595	11430	151		75.6	-68.60	-13.39	
155-GC, 70 cm	GC	Mussel Cliff	152°35.385'	3°19.201'	1595	12889	213		60.5	-57.08	-14.75	
155-GC, 95 cm	GC	Mussel Cliff	152°35.385'	3°19.201'	1595	13023	111		117	-56.91		
063_ROV-01, 0-5 cm	PC	Mussel Cliff	152°35.411'	3°19.079'	1539	55300	798	25.9	67.1	-51.30	-16.65	-13.10
063_ROV-01, 5-10 cm	PC	Mussel Cliff	152°35.412'	3°19.079'	1539	41449	1035	57.2	37.9	-51.10	-17.15	-17.12
063_ROV-01, 10-15 cm	PC	Mussel Cliff	152°35.413'	3°19.079'	1539	33825	294	13.8	110	-41.40	-17.91	-11.96

WD – water depth, BW – bottom water, PW – pore water, Nis – Niskin bottle, MS – Major Sampler, PC – push core, GC – gravity core, ppmV – concentration in the headspace

Figure S7: Stable carbon isotope composition of methane (C1), ethane (C2) and propane (C3). Warm colors (yellow-orange-red-brown) correspond to samples (headspace and dry gas) from Karambusel, cool colors (blue-grey) to samples from Mussel Cliff. All samples from Mussel Cliff show a normal carbon isotope behaviour, i.e. they are becoming enriched in ^{13}C (less negative $\delta^{13}C$) with increasing carbon number (C_x). Also some samples from Karambusel show this pattern. However, many Karambusel samples show a ‚reversal', meaning that carbon isotopes of ethane (C_2) are heavier (more enriched in ^{13}C) than those of propane (C_3).

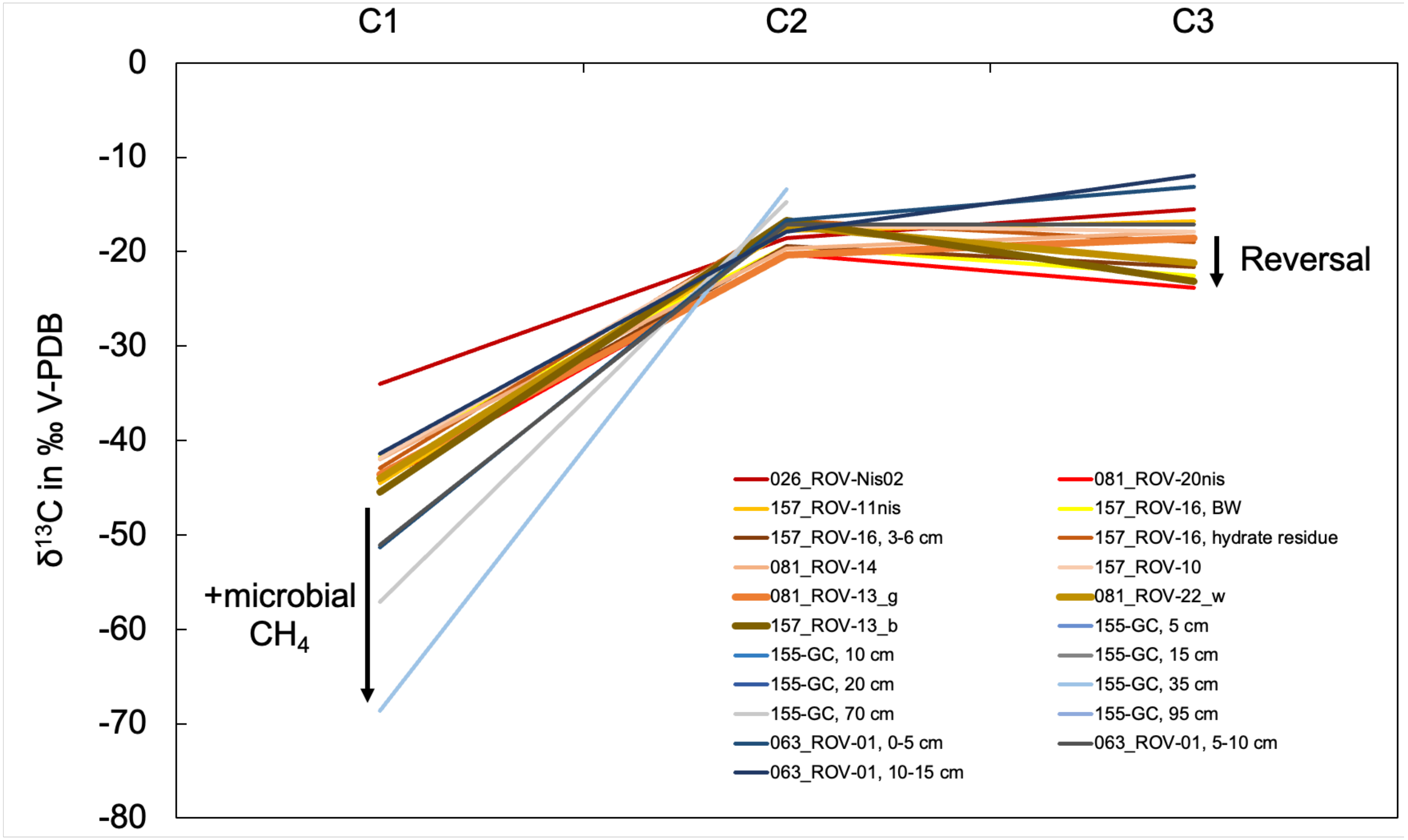

Figure S8: Sulfide-rich samples from Mussel Cliff. A) Highly porous (~30 % pore space; sample 162_TVG-04) to B) semi-massive (sample 161_TVG-03) hand specimen dominated by pyrite and marcasite almost completely replacing shell fragments and other biogenic matter. C) Pyritized shell fragments. D) Semi-massive pyrite and marcasite matrix cementing and replacing shells, worms, and other biogenic matter. E) Pore space or fluid channel rimmed by colloform pyrite/marcasite layers with abundant pyrite framboids.

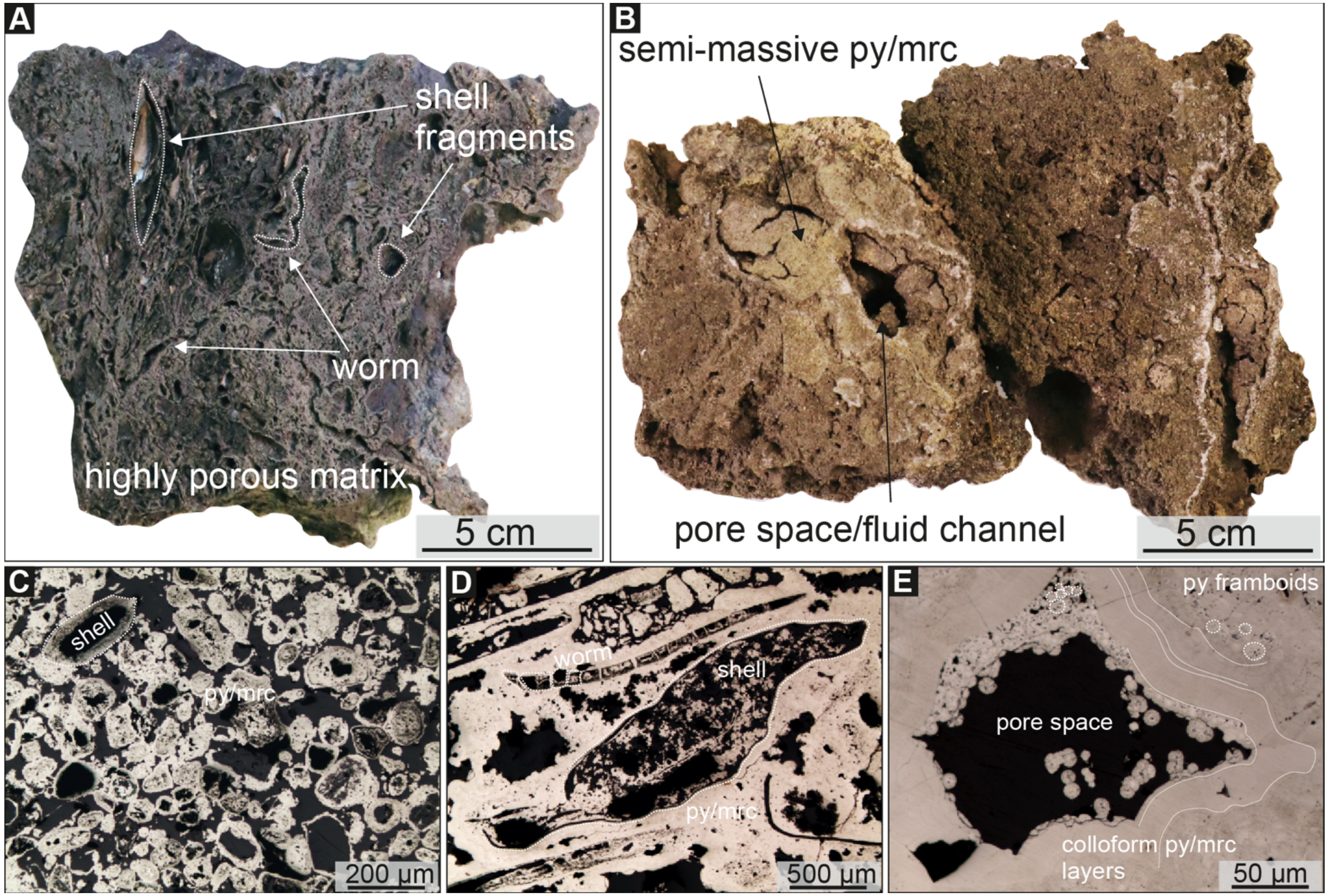

Table S9: U-Th age constraints of sulfides collected at the top of Conical Seamount.

Sample	^{232}Th	^{230}Th	Uncertainty	$^{234}U/^{238}U$	Uncertainty	^{234}U	Uncertainty	$^{230}Th/^{234}U$	Uncertainty	Age	Uncertainty
	µg g^{-1}	dpm g^{-1}	dpm g^{-1}			dpm g^{-1}	dpm g^{-1}			ka	ka
SO133/2 au-Au-2	0.0458	0.00270	±0.000080	1.1300	±0.0524	0.004580	±0.000151	0.5910	±0.0630	94.5	+10.4/-8.6
SO133/2 25-5C3	bql.	0.00433	±0.000127	1.1600	±0.0710	0.007090	±0.000306	0.6120	±0.0568	99.4	+11.2/-8.9
SO133/2 40-py	0.0396	0.00428	±0.000084	1.1700	±0.0672	0.004220	±0.000172	1.0100	±0.0803	>300	
SO133/2 25-8c6	bql.	0.00614	±0.000106	1.0900	±0.0604	0.011100	±0.000433	0.5540	±0.0865	86.2	+9.4/-7.9
mean										93.4±6.7	

bql. – below quantification limit, dpm g^{-1} – decay per minute and per gram

Please note: This data has been acquired by Dr. Jan Scholten at the Institute of Geosciences, Kiel University, in the late 1990ies. The data were released in the final report of the SO133 Project (BMBF 03G0133) but never got published. Dr. Scholten is retired since 2023 but made his data available to our study.
